# Total Hip Replacement for the Treatment of End Stage Arthritis of the Hip: A Systematic Review and Meta-Analysis

**DOI:** 10.1371/journal.pone.0099804

**Published:** 2014-07-08

**Authors:** Alexander Tsertsvadze, Amy Grove, Karoline Freeman, Rachel Court, Samantha Johnson, Martin Connock, Aileen Clarke, Paul Sutcliffe

**Affiliations:** Warwick Evidence, Division of Health Sciences, Warwick Medical School, The University of Warwick, Coventry, England; University of Thessaly School of Medicine, Greece

## Abstract

**Background:**

Evolvements in the design, fixation methods, size, and bearing surface of implants for total hip replacement (THR) have led to a variety of options for healthcare professionals to consider. The need to determine the most optimal combinations of THR implant is warranted. This systematic review evaluated the clinical effectiveness of different types of THR used for the treatment of end stage arthritis of the hip.

**Methods:**

A comprehensive literature search was undertaken in major health databases. Randomised controlled trials (RCTs) and systematic reviews published from 2008 onwards comparing different types of primary THR in patients with end stage arthritis of the hip were included.

**Results:**

Fourteen RCTs and five systematic reviews were included. Patients experienced significant post-THR improvements in Harris Hip scores, but this did not differ between impact types. There was a reduced risk of implant dislocation after receiving a larger femoral head size (36 mm vs. 28 mm; RR = 0.17, 95% CI: 0.04, 0.78) or cemented cup (vs. cementless cup; pooled odds ratio: 0.34, 95% CI: 0.13, 0.89). Recipients of cross-linked vs. conventional polyethylene cup liners experienced reduced femoral head penetration and revision. There was no impact of femoral stem fixation and cup shell design on implant survival rates. Evidence on mortality and complications (aseptic loosening, femoral fracture) was inconclusive.

**Conclusions:**

The majority of evidence was inconclusive due to poor reporting, missing data, or uncertainty in treatment estimates. The findings warrant cautious interpretation given the risk of bias (blinding, attrition), methodological limitations (small sample size, low event counts, short follow-up), and poor reporting. Long-term pragmatic RCTs are needed to allow for more definitive conclusions. Authors are encouraged to specify the minimal clinically important difference and power calculation for their primary outcome(s) as well CONSORT, PRISMA and STROBE guidelines to ensure better reporting and more reliable production and assessment of evidence.

## Introduction

Over the past few decades, total hip replacement (THR) has been reported as clinically effective in treating pain and disability resulting from late stage arthritis of the hip [1]. THR is indicated for patients who failed to respond to non-surgical management options such as pharmaceutical treatments (e.g., analgesics, anti-inflammatory agents, steroid injections, topical treatments), self-management, patient education, acupuncture, exercise, physical therapy, or manual therapy [2]–[3]. This procedure involves the replacement of a damaged hip joint with an artificial hip prosthesis consisting of an acetabular cup (with or without shell) a femoral stem, and femoral head.

Rates of THR in the western world have steadily increased between 2005 and 2010 [3]. A total of 86,488 hip procedures were recorded on the UK National Joint Registry in 2012; a 7.5% increase from 2011 [4]. In 2012, 76,448 primary hip procedures were undertaken and 10,040 revisions. This ‘revision’ burden now stands at 12% of total hip activity compared to 11% in 2011 [4].

Continuing marketing approval for evolving design of implant components, of prosthesis to bone fixation methods (e.g., cemented, cementless, hybrid), of prosthesis femoral head size, and of bearing surface articulations (e.g., metal, ceramic, polyethylene) has resulted in a multitude of options for care providers and patients.

This systematic review aimed to evaluate the evidence on the clinical effectiveness of different types of THR used in the treatment of pain and disability in people with end stage arthritis of the hip.

## Materials and Methods

This systematic review forms part of independent research commissioned by the National Institute for Health Research (project number 11/118); the full protocol and guidance is accessible from: http://www.nice.org.uk.

### Search strategy

Searches were undertaken in December 2012 and were date-limited from 2008. Electronic searches were conducted in MEDLINE, MEDLINE In-Process, Embase, Science Citation Index, Cochrane Library (Cochrane Database of Systematic Reviews and Cochrane Central Register of Controlled Trials), Current Controlled Trials, ClinicalTrials.gov, Database of Abstracts of Reviews of Effectiveness (DARE), and HTA databases. Reference lists and websites of hip implant manufacturers and major orthopaedic organisations were screened for relevant publications. Details of MEDLINE and Embase searches are presented in Appendix supporting information File S1. Searches were adapted for other databases.

### Study eligibility criteria

Full text English-language reports of RCTs and systematic reviews comparing different types of primary THR were eligible for inclusion. The population included patients with end stage hip arthritis for whom non-surgical management has failed. The THR types were compared on the composition/material, design, bearing surface, fixation method, and size of components (acetabular cup, femoral stem, and femoral head). Non-RCTs, cohort studies, economic evaluations, editorials, letters, and conference abstracts were excluded. Studies focusing on indications other than end stage arthritis of the hip, on revision surgery, on hip resurfacing or those comparing different THR operative approaches (e.g., mini-incision vs. standard-incision) were also excluded.

We further limited our inclusion to studies with sample size of 100 participants or more. This was done in order to minimize evidence with inconclusive, i.e., uninformative results (i.e., statistically non-significant effect estimates with wide 95% confidence intervals). Based on our calculations, the sample size of 100 was the minimum sample for a study which would have 90% power (two-tailed test significance level of 0.05) to detect the mean difference of at least 10 points on the Harris Hip score (with standard deviation of 15 based on external sources) [5]–[6].

### Outcomes of interest

Primary outcome measures were measures of hip function and symptoms (Harris Hip; [7] Oxford Hip; [8] Western Ontario and McMaster University Osteoarthritis Index [WOMAC] [9]), all-cause mortality; risk of revision (or implant survival rate); and femoral head penetration rate. Secondary outcomes included other validated clinical/functional measures (McMaster-Toronto Arthritis patient Preference Disability Questionnaire [MACTAR] [10], Merle D'Aubigne Postel [11], University of California Los Angeles [UCLA] activity score [12], health-related quality of life [HRQOL] measures), and peri/post-procedural complications (i.e., implant dislocation, infection, osteolysis, aseptic loosening, femoral fracture, and deep vein thrombosis).

### Study selection and data extraction

Two independent reviewers screened all bibliographic records for title/abstract and then for full text. Reasons for exclusion of full text papers were documented in the study flow diagram [13]. The same reviewers independently extracted relevant data which was then cross-checked. Disagreements were resolved by discussion and with a third reviewer. The extracted data included study, participant, intervention/comparator (types of THR, basis of comparison, operator skill), and outcome characteristics. If data permitted, we attempted to calculate missing statistical parameters (e.g., risk ratios, mean differences, and 95% confidence intervals). For individual studies with zero events in one or both treatment arms, risk ratios and 95% confidence intervals (95% CIs) were not estimated. The 95% CIs and standard errors were used to derive standard deviations or vice versa. All calculated parameters were entered into the data extraction sheets.

### Assessment of risk of bias (ROB) and methodological quality

Two reviewers independently assessed ROB of RCTs and methodological quality of systematic reviews using the Cochrane Collaboration ROB tool [14] and the AMSTAR tool [15], respectively.

The Cochrane ROB tool [14] addresses threats to several internal validity domains (selection, performance, detection, attrition, reporting, and other pre-specified bias). The ROB for performance, detection, and attrition bias was assessed for *a priori* defined groups of objective and subjective outcomes separately and was classified as high, low, or unclear. Afterwards, for each RCT, within-study summary ROB rating was derived for subjective and objective outcomes. At data synthesis stage (evidence grading), the across-study average summary ROB was determined and assigned to each outcome of interest.

The AMSTAR tool [15] covers domains of research question, inclusion/exclusion criteria, search strategy, data extraction, ROB assessment, heterogeneity, and publication bias. For convenience of presentation, the quality of each SR was rated according to the number of items satisfied: high (range: 9–11), medium (range: 5–8), and low (range: 0–4).

### Meta-analysis

The decision to pool study results was based on degree of similarity in the methodological and clinical characteristics of studies under consideration. Estimates of post-treatment mean difference (MD) for continuous outcomes and risk ratios (RR) for binary outcomes (except for rare events) were pooled using a random-effects model [16]. Dichotomous outcomes with low event rates (5.0%–10.0%) were pooled as RR using Mantel-Haenszel (MH) fixed-effect models. Dichotomous outcomes for studies with very low event rates (≤5.0%) or zero events in one of the treatment arms were pooled as odds ratio (OR) using Peto fixed-effect model [17]. The heterogeneity was assessed through inspection of forest plots, Cochran's Q and I^2^ statistics, and was judged according to pre-determined levels of statistical significance (Chi-square p<0.10 and/or I^2^>50%).

### Other analyses

Publication bias was planned to be examined by visual inspection of asymmetry and regression tests on funnel plots [18]. Clinical and methodological sources of statistical heterogeneity was planned to be explored through *a priori* defined subgroup and sensitivity analyses (age, gender, activity levels, duration of follow-up, risk of bias items).

### Grading overall quality of clinical effectiveness evidence

The overall quality of evidence for each gradable outcome was assessed using the system developed by Grading of Recommendations, Assessment, Development, and Evaluation (GRADE) Working Group system (http://www.gradeworkinggroup.org). This approach [19] indicates levels of confidence in the observed treatment effect(s) and categorizes the evidence for each outcome into high, moderate, low, or very low grade based on the summary ROB across studies, consistency (heterogeneity), directness (applicability), precision, and publication/reporting bias. Gradable outcomes were Harris Hip score, WOMAC score, revision, mortality, femoral head penetration, and implant dislocation.

### Evidence synthesis and interpretation

Comparison and synthesis of results for each outcome of interest were summarised and categorised as conclusive (either ‘there is difference’ or ‘there is no difference’) or inconclusive (indeterminate results due to statistical uncertainty, statistical heterogeneity/inconsistency in treatment effects, and/or incomplete information). This conclusion was based on statistical significance of the observed difference, magnitude of the effect estimate, width of the 95% CIs, whether the 95% CI included a minimal clinically important difference (MCID) for a given outcome, and consistency in terms of effect direction and statistical significance. We ascertained the MCIDs for clinical/functional measures such as Harris hip score (MCID range: 7–10), Oxford hip score (MCID range: 5–7), WOMAC score (MCID: 8), and EQ-5D (MCID: 0.074) from previous empirical research evidence [6], [20]–[21].

## Results

Our searches identified 1,523 unique records, of which, 27 were included in this review [22], [23] (This piece of information contains information from a study with multiple publications [66] (See Table S1 in File S1)), [24]–[48]. Four RCTs were represented by multiple publications and the review cites them as Bjorgul 2010 [22], Engh 2012 [26] [This piece of information contains information from a study with multiple publications [69] (See Table S1 in File S1)], Capello 2008 [28] [This piece of information contains information from a study with multiple publications [70] (See Table S1 in File S1)], and Corten 2011 [32].

**Figure 1:**
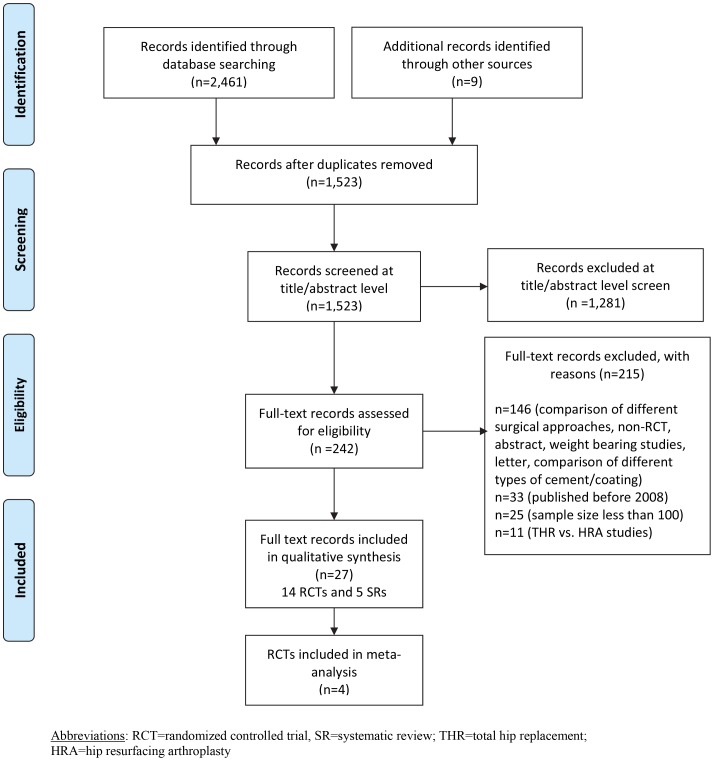
PRISMA study flow diagram.

Thus, the review included 14 RCTs [22], [24]–[26], [28], [32], [36]–[43] and five systematic reviews [44]–[48]. The study flow diagram is given in Figure 1 and Checklist S1. Please see Table S20 in File S1 for full details of the systematic reviews. [The reviews contain information from studies with multiple publications [80], [81] (See Table S20 in File S1)].

### RCTs

#### Study characteristics

Included RCTs compared evidence on clinical effectiveness between different types of THR based on the composition [40], design [28], [41], bearing surface [25]–[26], [28], [37]–[39], [43], fixation method [22], [24], [32], [42], and size [36] of implant components (Table 1) [The studies contain information from multiple publications [28]– (See Table S1 in File S1)]. RCTs were conducted in the USA, the UK, Australia, Norway, Serbia, South Korea, and Canada. [Please see Table S1 in File S1 for full details of the RCT studies [23], [24], [26], [28], [29], [30], [31], [33], [43], [67]–[79]].

**Table 1:** Randomized controlled trials according to basis of hip implant comparison.

Basis of comparison	Study ID
**Cup fixation** (cemented vs. cementless)	Bjorgul 2010 [22]
	Angadi 2012 [24]
**Cup liner bearing surface** (XLPE vs. non-XLPE)	McCalden 2009 [25]
	Engh 2012 [26]
**Cup shell design** (porous-coated vs. arc-deposited HA-coated)	Capello 2008 [28]
**Cup and femoral stem fixation** (cemented vs. cementless)	Corten 2011 [32]
**Femoral head size** (36 mm vs. 28 mm)	Howie 2012 [36]
**Femoral head bearing** (oxinium vs. CoCr)	Lewis 2008 [37]
**Femoral head-on-cup liner bearing**	
Ceramic-on-ceramic vs. ceramic-on-PE	Amanatullah 2011 [38]
Ceramic-on-ceramic vs. CoCr-on-PE	Capello 2008 [28]
Steel-on-PE vs. CoCr-on-PE vs. oxinium-on-PE vs. CoCr-on-XLPE vs. oxinium-on-XLPE	Kadar 2011 [39]
Ceramic-on-ceramic vs. CoCr-on-XLPE	Bascarevic 2010 [43]
**Femoral stem composition** (CoCr vs. titanium)	Healy 2009 [40]
**Femoral stem design** (short metaphyseal-fitting vs. conventional metaphyseal- and diaphyseal-filling)	Kim 2011 [41]
**Femoral stem fixation** (cemented vs. cementless)	Kim 2011 [42]

XLPE = cross-linked polyethylene; PE = polyethylene; HA = hydroxyapatite; CoCr = cobalt chrome.

Maximum length of follow-up was 20 years [32], [42]. The mean age in individual studies ranged from 45 [42] to 72 years [25], [36] and the proportion of women ranged from 24% [42] to 75% [43]. The mean follow up period of included studies is 8.4 years with a range of 1 [74] to 20 [33], [71]–[73], [79] years. Participant baseline characteristics are given in File S1, and Table S1 in File S1.

#### Risk of bias

Overall, five (36%) and eight (57%) RCTs reported an adequate method for random sequence generation and treatment allocation concealment respectively (low ROB). RCTs had lower risks of performance and detection bias for objective (e.g., mortality, dislocation) vs. subjective (e.g., functional scores) outcomes (92%–100% vs. 15%–23%). Most RCTs failed to report the blinding status of patients, study personnel, and/or outcome assessors. Attrition bias was judged at low risk for at least eight RCTs (57%). Five RCTs (36%) were at high risk of selective reporting of outcome. Risk of other bias (e.g., funding source, baseline imbalance, inappropriate analysis) was rated as high for about one third of the RCTs. See the ROB assessment for the included RCTs (File S1 and Table S2 and Figure S1 in File S1).

#### Synthesis of evidence on clinical effectiveness

Outcome-specific results are provided in Appendix Tables (File S1 and Tables S3–S18 in File S1).

To render outcome reporting bias and consistency criteria applicable for grading, only THR comparison categories which included at least two studies (cup fixation: cemented vs. cementless; cup liner surface: cross-linked polyethylene [XLPE] vs. [non-XLPE]) were selected. The overall quality grade for gradable outcomes was very low/low (for WOMAC, revision, mortality), moderate (for Harris Hip score, femoral head penetration), and high (for implant dislocation). See the results for graded outcomes (File S1 and Table S19 in File S1).

**Figure 2:**
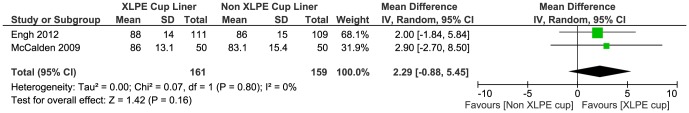
Mean post Harris hip score measured at follow up.

Summary results are provided in Table 2. Across seven studies, the mean post-THR Harris Hip score measured at different follow-ups (6 months to 10 years) did not differ between the THR groups of cup fixation (cemented vs. cementless; moderate grade) [22], [24], cup liner surface (XLPE vs. traditional polyethylene [PE]; moderate grade; pooled MD = 2.29, 95% CI: −0.88, 5.45) [Figure 2] [25]–[26], cup and stem fixation (cemented vs. cementless) [32], and femoral head-on-cup articulation (metal/oxinium-on-XLPE vs. metal/oxinium-on-PE [39]; ceramic-on-ceramic vs. metal-on-XLPE [43]). Similarly, there were no differences in WOMAC and Short Form (SF)-12 scores between the THR groups of XLPE vs. traditional PE cup liners; very low grade [25] as well as in MACTAR and Merle D'Aubigne Postel scores between the THR groups of cup and femoral stem fixation (cemented vs. cementless) [32].

**Table 2:** Summary of evidence regarding the differences between the compared types of THR for each reported outcome (randomized controlled trials).

Conclusive evidence	Conclusive evidence	Inconclusive evidence
Difference	No difference	
***Cup fixation Cemented vs. Cementless *** [*22*], [*24*]	***Cup fixation Cemented vs. Cementless *** [*22*], [*24*]	***Cup fixation Cemented vs. Cementless *** [*22*], [*24*]
Implant dislocation [high grade evidence] [22], [24] **In favor of cemented**	Harris Hip score[moderate grade evidence] [22], [24] Implant survival [22], [24]	Mortality [very low grade evidence] [22] Revision [very low grade evidence] [24] Osteolysis [24] Aseptic loosening [24] Infection [24]
***Cup liner bearing surface XLPE vs. Non XLPE *** [*25*], [*26*]	***Cup liner bearing surface XLPE vs. Non XLPE *** [*25*], [*26*]	***Cup liner bearing surface XLPE vs. Non XLPE *** [*25*], [*26*]
Femoral head penetration [moderate grade evidence] [25], [26] Revision rate [very low grade evidence] [26] **In favor of XLPE**	Harris Hip score [moderate grade evidence] [25], [26] WOMAC score [very low grade evidence] [25] SF-12 (mental/physical) [25]	Mortality [low grade evidence] [25], [26] Implant survival [26] Osteolysis [25], [26] Aseptic loosening [26] Femoral fracture [26]
***Cup shell design Porous-coated vs. Arc-deposited HA-coated *** [*28*]	***Cup shell design Porous-coated vs. Arc-deposited HA-coated *** [*28*]	***Cup shell design Porous-coated vs. Arc-deposited HA-coated *** [*28*]
None	Implant survival	Harris Hip score, Revision, Implant dislocation, Osteolysis, Femoral fracture
***Cup and femoral stem fixation Cemented vs. Cementless *** [*32*]	***Cup and femoral stem fixation Cemented vs. Cementless *** [*32*]	***Cup and femoral stem fixation Cemented vs. Cementless *** [*32*]
Survival rate **In favor of cementless**	Harris Hip score, Merle D'Aubigne Postel score, MACTAR score	WOMAC score, Mortality, Revision, Aseptic loosening
***Femoral head size 36 mm vs. 28 mm*** [*36*]	***Femoral head size 36 mm vs. 28 mm*** [*36*]	***Femoral head size 36 mm vs. 28 mm*** [*36*]
Implant dislocation **In favor of 36 mm**	None	Mortality, Revision
***Femoral head bearing surface Oxinium vs. CoCr *** [*37*]	***Femoral head bearing surface Oxinium vs. CoCr *** [*37*]	***Femoral head bearing surface Oxinium vs. CoCr *** [*37*]
None	None	Harris Hip score, SF-12, WOMAC score, Implant survival, Revision, Implant dislocation, Aseptic loosening, Infection
***Femoral head-on-cup liner bearing-I Ceramic-on-Ceramic vs. Metal-on-PE *** [*28*]	***Femoral head-on-cup liner bearing-I Ceramic-on-Ceramic vs. Metal-on-PE *** [*28*]	***Femoral head-on-cup liner bearing-I Ceramic-on-Ceramic vs. Metal-on-PE *** [*28*]
Osteolysis **In favor of ceramic-on-ceramic**	None	Harris Hip score, Revision, Implant dislocation
***Femoral head-on-cup liner bearing-II Ceramic-on-Ceramic vs. Ceramic-on-PE *** [*38*]	***Femoral head-on-cup liner bearing-II Ceramic-on-Ceramic vs. Ceramic-on-PE *** [*38*]	***Femoral head-on-cup liner bearing-II Ceramic-on-Ceramic vs. Ceramic-on-PE *** [*38*]
None	None	Harris Hip score, SF-12, Revision, Implant dislocation, Osteolysis, Infection, Deep vein thrombosis
***Femoral head-on-cup liner bearing-III Steel-on-PE vs. CoCr/Oxinium-on-XLPE vs. CoCr/Oxinium-on-PE *** [*39*]	***Femoral head-on-cup liner bearing-III Steel-on-PE vs. CoCr/Oxinium-on-XLPE vs. CoCr/Oxinium-on-PE *** [*39*]	***Femoral head-on-cup liner bearing-III Steel-on-PE vs. CoCr/Oxinium-on-XLPE vs. CoCr/Oxinium-on-PE *** [*39*]
Femoral head penetration **In favor of Steel-on-PE or CoCr/Oxinium-on-XLPE**	Harris Hip score	None
***Femoral head-on-cup liner bearing surfaces–IV Ceramic-on-Ceramic vs. CoCr-on-XLPE *** [*43*]	***Femoral head-on-cup liner bearing surfaces–IV Ceramic-on-Ceramic vs. CoCr-on-XLPE *** [*43*]	***Femoral head-on-cup liner bearing surfaces–IV Ceramic-on-Ceramic vs. CoCr-on-XLPE *** [*43*]
None	Harris Hip score	Revision, Implant dislocation, Infection, Deep vein thrombosis
***Femoral stem composition CoCr vs. Titanium *** [*40*]	***Femoral stem composition CoCr vs. Titanium *** [*40*]	***Femoral stem composition CoCr vs. Titanium *** [*40*]
None	None	Harris Hip score, Implant survival, Revision, Implant dislocation, Osteolysis, Aseptic loosening, Femoral fracture, Infection
***Femoral stem design Short metaphyseal-fitting vs. Conventional metaphyseal- and diaphyseal-filling *** [*41*]	***Femoral stem design Short metaphyseal-fitting vs. Conventional metaphyseal- and diaphyseal-filling *** [*41*]	***Femoral stem design Short metaphyseal-fitting vs. Conventional metaphyseal- and diaphyseal-filling *** [*41*]
None	None	Harris Hip score, Mortality, Revision
***Femoral stem fixation Cemented vs. Cementless *** [*42*]	***Femoral stem fixation Cemented vs. Cementless *** [*42*]	***Femoral stem fixation Cemented vs. Cementless *** [*42*]
None	Implant survival	Harris Hip score, UCLA score, WOMAC score, Revision, Osteolysis

XLPE = cross-linked polyethylene; PE = polyethylene; HA = hydroxyapatite; CoCr = cobalt chrome; WOMAC = Western Ontario and McMaster University Osteoarthritis Index; SF-12 = Short Form Health Survey; RCT = randomized controlled trial; UCLA = University of California, Los Angeles activity scale.

**Figure 3:**
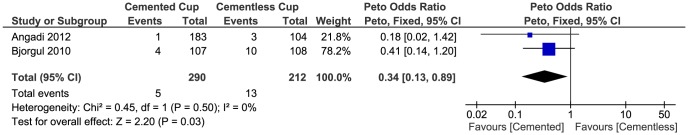
Implant dislocation of cemented cup vs. cementless cup.

There was a reduced risk of implant dislocation with use of cemented cup (vs. cementless cup; high grade; pooled OR = 0.34, 95% CI: 0.13, 0.89) (Figure 3) [22], [24] or larger femoral head size (36 mm vs. 28 mm) [36]. In three other RCTs, patients who received THR with XLPE cup liners experienced reduced femoral head penetration rate (moderate grade evidence) [25]–[26], [39] and risk of revision (risk ratio: 0.18, 95% CI: 0.04, 0.78; very low grade evidence) [26] compared to recipients of conventional PE cup liners. The recipients of ceramic-on-ceramic articulations (vs. metal-on-PE) had a reduced risk of osteolysis [28]. Although, in one trial, the use of cementless fixation of cup and femoral stem (vs. cemented) was associated with better implant survival rate [32], other trials showed no apparent impact of cup [22], [24] or femoral stem [42] fixation (cemented vs. cementless) and cup shell design (porous-coated vs. arc-deposited HA-coated) [28] on implant survival rates.

**Figure 4:**
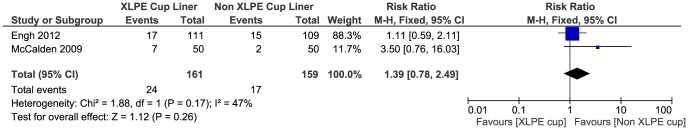
Evidence of revision.

Evidence on revision [24], [28], [32]–[38], [40]–[43], the UCLA score [42], mortality (very low-to-low grade; pooled RR = 1.39, 95% CI: 0.78, 2.49) [Figure 4] [22], [25]–[26], [32], [36], [41], aseptic loosening [24], [26], [32], [37], [40], femoral fracture [26], [28], [40], infection [24],[37]–[38],[40],[43], and deep vein thrombosis [38], [43] was inconclusive. Also, the evidence for all outcomes reported in four studies was rendered inconclusive (very low grade evidence) [37]–[38], . Results were considered inconclusive due to partial reporting (missing data to allow for effect estimates, confidence intervals, standard errors, standard deviations, p-values), great uncertainty (wide confidence intervals), zero event counts, and/or inconsistency in estimates (Table 2).

### Systematic reviews

Five systematic reviews evaluated the effectiveness of THRs (see Table S20 in File S1) according to cup fixation methods (cemented vs. cementless) [44]–[46] and implant articulations [47]–[48] on post-operative functional scores (Harris Hip score, Oxford Hip score) [44]–[45], [47], risk of revision, and implant survival rate [45]–[46]. Searches in the systematic reviews were undertaken between July 2007 [48] and June 2011 [46].

The methodological quality of the five systematic reviews is presented in File S1 (and Table S21 in File S1). Two systematic reviews [44], [47] were of high quality (AMSTAR score range of: 9–10) and two systematic reviews [45], [48] were of medium quality (AMSTAR score range of: 5–7). The one remaining systematic reviews [46] had a low quality (AMSTAR score: 4) because of inappropriate analysis, absence of duplicate study selection, limited literature search, failure to address publication bias, and lack of information on conflict of interest.

The outcome-specific and summary evidence results for the systematic reviews [44]–[48] are provided in File S1 (and Tables S22–S29 in File S1) and Table 3, respectively. Most evidence was rendered inconclusive due to unreported pooled results across RCTs (i.e., only narrative synthesis), inappropriate pooling methods (e.g., indirect naïve comparison of single group cohorts; pooling of studies of different design) [45]–, or inconsistent summary findings [47]. One review indicated no difference in the risk of revision between zirconium-on-polyethylene vs. non zirconium-on-polyethylene articulations [48].

**Table 3:** Summary of evidence regarding the differences between the compared types of THR for each reported outcome (Systematic Reviews).

Conclusive evidence	Conclusive evidence	Inconclusive evidence
Difference	No difference	
***Cup fixation Cemented vs. Cementless *** [*44*], [*46*]	***Cup fixation Cemented vs. Cementless *** [*44*], [*46*]	***Cup fixation Cemented vs. Cementless *** [*44*], [*46*]
None	None	Harris Hip score [44], [45] Oxford Hip score [44], Revision [45], [46], [65] Implant survival [45], [46] Implant dislocation [46] Osteolysis [46], [66] Aseptic loosening [46]
***Femoral head-on-cup liner bearing Different comparisons*** * [*47*], [*48*]	***Femoral head-on-cup liner bearing Different comparisons*** * [*47*], [*48*]	***Femoral head-on-cup liner bearing Different comparisons*** * [*47*], [*48*]
None	Revision [48]	Harris Hip score [47], SF-12 [47] Revision [47] Implant dislocation [47]

PE = polyethylene.

*Metal-on-Metal vs. Metal-on-PE [47].

Ceramic-on-Ceramic vs. Ceramic-on-PE [47].

Ceramic-on-PE vs. Metal-on-PE [47].

Metal-on-Metal vs. Ceramic-on-Ceramic [47].

Zirconia-on-PE vs. Non Zirconia-on-PE [48].

### Publication bias and heterogeneity

The extent of publication bias could not be explored due to insufficient numbers of data points in the forest/funnel plots. The data from RCTs was too sparse and heterogeneous (in terms of different types of THRs) to allow for the exploration of whether study-level methodological or patient-related characteristics influenced treatment effects. None of the included RCTs reported within-study subgroup treatment effects.

## Discussion

The large proportion of evidence summarised in this review was inconclusive due to poor reporting, missing data, inconsistent results, and/or great uncertainty in the treatment effect estimates. The majority of studies suggested significantly improved post-surgery scores for functional and clinical measures (Harris Hip, Oxford Hip, WOMAC, MACTAR, Merle D'Aubigne Postel, and SF-12) in participants regardless of the type of THR they received. Most evidence indicated no difference for these measures between different types of THR. There was a reduced risk of implant dislocation for participants receiving THR with a larger femoral head size (vs. smaller head size) or with cemented cup (vs. cementless; high grade evidence). Moreover, the evidence suggested reduced femoral head penetration rate and risk of implant revision for participants who received cross-linked polyethylene vs. conventional polyethylene cup liner bearings. Participants with ceramic-on-ceramic articulations (vs. metal-on-polyethylene) experienced reduced risk of osteolysis.

The limitations of the evidence warrant cautious interpretation of the findings. Great uncertainty in treatment effect estimates and incomplete reporting rendered some of the evidence inconclusive. The evidence on complications was scarce. It is unclear whether this is due to the absence or rarity of these events or it is simply due to under reporting. In light of poor reporting, it was not possible to explore contextual factors which might have influenced study results. For example, the lack of blinding of participants and study personnel may have led to systematic differences in care giving or co-interventions across implant groups which would independently influence outcome measures. None of the studies reported the experience levels and skills of study personnel and care givers. Any imbalance between study treatment groups in these factors may have influenced participants' prognosis independently of treatment. Systematic differences in the maturity of any given implant technology may have additionally influenced the observed treatment effects [49]–[53]. The paucity of data hindered the exploration of variation in treatment effect across subgroups of patients or methodological features of RCTs. Apart from limitations of the evidence itself, we limited the scope of this review to evidence published in English in 2008 or later. However, note that systematic reviews would provide the summary evidence for individual studies published before 2008. We limited our focus on studies with sample size of 100 or more participants. Since this limitation was not dependant on statistical significance (i.e., smaller studies were excluded regardless of statistical significance of their effect estimates), the effect of selection bias is less likely. Moreover, it has been empirically shown that inclusion of smaller studies may bias the observed treatment benefit upwards due to phenomena called ‘small study effect’ [54]–[57].

The poor reporting reduces the applicability of the findings to routine clinical practice in the UK. Generally, most studies were conducted in the Western world and reported patient-oriented as well as other important outcomes (e.g., revision, survival, mortality, complications) representative of those measured in clinical practice. The proportion of patients with primary osteoarthritis across the majority of studies was 60% or greater.

Auto alerts of searches set up to capture relevant articles published after the dates of the searches identified three new relevant systematic reviews which compared the effectiveness of THR using different articulations (metal-on-metal vs. metal-on-polyethylene) [58], implant fixation methods (cemented vs. cementless) [59], or femoral stem coating materials (hydroxyapatite-coated vs. non-hydroxyapatite-coated) [60]. Outcomes measured were risk of revision, Harris Hip score, mortality, and complications. In agreement with our findings, pooled estimates for post-surgery Harris Hip scores reported in all three systematic reviews showed no difference between THR groups. Pooled estimates for revision (6 RCTs; RR = 1.44, 95% CI: 0.88, 2.36), mortality (5 RCTs; RR = 1.06, 95% CI: 0.73, 1.52), and complications (4 RCTs; RR = 1.54, 95% CI: 0.21, 11.03) between THR groups with cemented vs. cementless fixation methods were statistically non-significant in one systematic review with wide 95% CIs (due to low event counts and small sample size of trials) compatible with a moderate-to-large effect size in either direction, rendering these findings inconclusive [59]. The pooled result from another systematic review [58] showed a greater risk of complications in the metal-on-metal vs. metal-on-polyethylene articulation group (3 RCTs; OR = 3.37, 95% CI: 1.57, 7.26).

Future large and long-term pragmatic RCTs are needed to replicate the findings of this review before more definitive conclusions are made. Study authors are encouraged to specify the minimal clinically important difference and power calculation for their primary outcome(s). This information would help to interpret the study findings both in terms of clinical and statistical terms. To improve the quality of reporting, authors are encouraged to conform to the recommendations outlined in the CONSORT (CONSOLIdated Standards of Reporting Trials) Statement [61] and its extension for RCTs evaluating non-pharmacologic interventions [62]. The recent CONSORT extension on patient-reported outcomes (PROs) would help to further improve the reporting quality of patient-reported functional and health quality outcome measures [63]. Use of the PRISMA (Preferred Reporting Items for Systematic Reviews and Meta-Analyses) [13] statement for reporting systematic reviews and meta-analyses and the STROBE (Strengthening the Reporting of Observation Studies in Epidemiology) [63] statement for reporting observational studies are also encouraged. Adequate reporting would facilitate more reliable assessment of evidence to inform health care decision makers, physicians, and patients regarding the selection of the most appropriate implants for particular patient groups.

In the absence of definitive findings from RCTs on the clinical effectiveness of different types of THR, patients and surgeons should probably consider observational data presented in the large National Registry reports; these are updated annually (e.g. UK NJR, Australian Registry, Swedish Registry), and hold data on important outcomes, notably revision rates, for tens to hundreds of thousands of patients who have received a variety of THR prostheses over one or more decades. Issa and Mont 2013 [64] point to the potential limitations of such large registries including: unequal distribution of measures that are included in the database, missing data for some patients, duplicated or unreported cases, delays in reporting, misclassification of outcomes, and also problems of showing causalities. However, in the absence of high quality randomised study reports as here, judicious consideration of Registry analyses may provide a better guide than inconclusive results from small RCTs of short duration. Nevertheless, well-designed clinical trials with appropriate power and follow-up are clearly preferred.

## Supporting Information

Checklist S1 **PRISMA study checklist.**
(DOCX)

File S1 **Figure S1, Table S1–S29.** Figure S1. Risk of bias graph for randomized controlled trials: review author's judgments about each risk of bias item. Table S1. Study and participant characteristics (randomized controlled trials). Table S2. Risk of bias summary table for randomized controlled trials: review author's judgments about each risk of bias item. Table S3. Harris Hip score (range: 0–100) *NB: Tables 3–18 (results for specific outcomes reported in randomized controlled trials).* Table S4. The Western Ontario and McMaster University Osteoarthritis Index (range: 0–100). Table S5. The McMaster-Toronto Arthritis Patient Preference Disability Questionnaire score (range: 0–30). Table S6. Merle D'Aubigne and Postel score (range: 0–18). Table S7. The University of California, Los Angeles activity scale (range: 1–10). Table S8. Short Form Health Survey (SF-12; range: 0–100). Table S9. Risk of revision (n/N). Table S10. Risk of mortality (n/N). Table S11. Femoral head penetration rate (mm/year). Table S12. Implant survival rate (%). Table S13. Risk of implant dislocation (n/N). Table S14. Risk of osteolysis (n/N). Table S15. Risk of aseptic loosening (n/N). Table S16. Risk of femoral fracture (n/N). Table S17.Risk of infection (n/N). Table S18. Risk of deep vein thrombosis (n/N). Table S19. GRADE evidence profile for gradable outcomes reported in randomized controlled trials (adapted from Guyatt et al., 2011)^19^. Table S20.Characteristics of included systematic reviews. Table S21. Methodological quality of systematic reviews (AMSTAR items). Table S22. Harris Hip score (range: 0–100) *NB - Tables 22–29 (results for each outcome reported in systematic reviews).* Table S23. Oxford Hip score (range: 0–48). Table S24. Short Form Health Survey (SF-12; range: 0–100). Table S25. Risk of revision (n/N). Table S26. Implant survival rate (%). Table S27. Risk of implant dislocation (n/N). Table S28. Risk of osteolysis (n/N). Table S29. Risk of aseptic loosening (n/N).
(DOCX)
