# Supplementary material for: Total Hip Replacement for the Treatment of End Stage Arthritis of the Hip: A Systematic Review and Meta-Analysis
Source: PLoS One. 2014 Jul 8;9(7):e99804. doi: 10.1371/journal.pone.0099804 (PMC4086719; doi:10.1371/journal.pone.0099804)
Supplement: File S1 — Figure S1, Table S1–S29. Figure S1. Risk of bias graph for randomized controlled trials: review author's judgments about each risk of bias item. Table S1. Study and participant characteristics (randomized controlled trials). Table S2. Risk of bias summary table for randomized controlled trials: review author's judgments about each risk of bias item. Table S3. Harris Hip score (range: 0–100) NB: Tables 3–18 (results for specific outcomes reported in randomized controlled trials). Table S4. The Western Ontario and McMaster University Osteoarthritis Index (range: 0–100). Table S5. The McMaster-Toronto Arthritis Patient Preference Disability Questionnaire score (range: 0–30). Table S6. Merle D'Aubigne and Postel score (range: 0–18). Table S7. The University of California, Los Angeles activity scale (range: 1–10). Table S8. Short Form Health Survey (SF-12; range: 0–100). Table S9. Risk of revision (n/N). Table S10. Risk of mortality (n/N). Table S11. Femoral head penetration rate (mm/year). Table S12. Implant survival rate (%). Table S13. Risk of implant dislocation (n/N). Table S14. Risk of osteolysis (n/N). Table S15. Risk of aseptic loosening (n/N). Table S16. Risk of femoral fracture (n/N). Table S17.Risk of infection (n/N). Table S18. Risk of deep vein thrombosis (n/N). Table S19. GRADE evidence profile for gradable outcomes reported in randomized controlled trials (adapted from Guyatt et al., 2011)19. Table S20.Characteristics of included systematic reviews. Table S21. Methodological quality of systematic reviews (AMSTAR items). Table S22. Harris Hip score (range: 0–100) NB - Tables 22–29 (results for each outcome reported in systematic reviews). Table S23. Oxford Hip score (range: 0–48). Table S24. Short Form Health Survey (SF-12; range: 0–100). Table S25. Risk of revision (n/N). Table S26. Implant survival rate (%). Table S27. Risk of implant dislocation (n/N). Table S28. Risk of osteolysis (n/N). Table S29. Risk of aseptic loosening (n/N). (DOCX) [file pone.0099804.s002.docx]

**Appendix 1:**

**Search strategy**

**Medline (1946 to October Week 4 2012) via OVID interface,** **searched on 05/11/2012**

| \| 1 \| exp Arthroplasty, Replacement, Hip/ \| 15246 \| \| --- \| --- \| --- \| \| 2 \| exp Hip Prosthesis/ \| 18304 \| \| 3 \| (tha or thr).tw. \| 23312 \| \| 4 \| exp Hip Joint/ \| 20108 \| \| 5 \| exp Hip/ \| 8480 \| \| 6 \| hip.tw. \| 79606 \| \| 7 \| ("femur head*" or "femoral head*" or acetabul*).tw. \| 20571 \| \| 8 \| exp Femur Head/ \| 7700 \| \| 9 \| exp Acetabulum/ \| 8243 \| \| 10 \| 4 or 5 or 6 or 7 or 8 or 9 \| 97057 \| \| 11 \| (arthroplast* or replace* or implant* or prosthes*).tw. \| 514865 \| \| 12 \| exp Joint Prosthesis/ \| 33736 \| \| 13 \| exp "Prostheses and Implants"/ \| 355910 \| \| 14 \| 11 or 12 or 13 \| 716289 \| \| 15 \| 10 and 14 \| 35876 \| \| 16 \| (surf* or resurf*).tw. \| 629176 \| \| 17 \| 10 and 16 \| 5573 \| \| 18 \| 1 or 2 or 3 or 15 or 17 \| 61490 \| \| 19 \| exp Arthritis, Rheumatoid/ or exp Arthritis/ \| 190095 \| \| 20 \| exp Osteoarthritis, Hip/ or exp Osteoarthritis/ \| 39813 \| \| 21 \| (arthrit* or osteoarthrit* or osteoarthrosis or "rheumatoid arthrit*").tw. \| 141102 \| \| 22 \| 19 or 20 or 21 \| 221909 \| \| 23 \| 18 and 22 \| 7739 \| \| 24 \| meta analysis.pt. \| 37222 \| \| 25 \| randomized controlled trial.pt. \| 340101 \| \| 26 \| (random* or "controlled trial*" or "clinical trial*" or rct).tw. \| 718263 \| \| 27 \| (metaanalys* or "meta analys*" or "meta-analys*").tw. \| 42924 \| \| 28 \| "systematic review*".tw. \| 34474 \| \| 29 \| 24 or 25 or 26 or 27 or 28 \| 846326 \| \| 30 \| 23 and 29 \| 614 \| \| 31 \| limit 30 to (english language and yr="2002 -Current") \| 443 \| |  |  |  |  |  |
| --- | --- | --- | --- | --- | --- | --- | --- | --- | --- | --- | --- | --- | --- | --- | --- | --- | --- | --- | --- | --- | --- | --- | --- | --- | --- | --- | --- | --- | --- | --- | --- | --- | --- | --- | --- | --- | --- | --- | --- | --- | --- | --- | --- | --- | --- | --- | --- | --- | --- | --- | --- | --- | --- | --- | --- | --- | --- | --- | --- | --- | --- | --- | --- | --- | --- | --- | --- | --- | --- | --- | --- | --- | --- | --- | --- | --- | --- | --- | --- | --- | --- | --- | --- | --- | --- | --- | --- | --- | --- | --- | --- | --- | --- | --- | --- | --- | --- | --- |
|  |  |  |  |  |  |
| **Embase 1974 to 2012 Week 44 via OVID interface, searched on 05/11/2012** |  |  |  |  |  |

| 1 | exp hip arthroplasty/ | 37471 |
| --- | --- | --- |
| 2 | exp total hip prosthesis/ | 20316 |
| 3 | (tha or thr).tw. | 25515 |
| 4 | exp hip/ | 29775 |
| 5 | hip*.tw. | 257572 |
| 6 | exp femur head/ | 9209 |
| 7 | exp acetabulum/ | 8120 |
| 8 | ("femoral head*" or "femur head*" or acetabul*).tw. | 25366 |
| 9 | 4 or 5 or 6 or 7 or 8 | 274870 |
| 10 | (arthroplast* or replace* or implant* or prosthes*).tw. | 661628 |
| 11 | exp joint prosthesis/ | 43247 |
| 12 | exp prosthesis/ or exp implant/ or exp "prostheses and orthoses"/ | 380676 |
| 13 | 10 or 11 or 12 | 872492 |
| 14 | 9 and 13 | 48019 |
| 15 | (surf* or resurf*).tw. | 809046 |
| 16 | 9 and 15 | 10412 |
| 17 | 1 or 2 or 3 or 14 or 16 | 80859 |
| 18 | exp arthritis/ or exp chronic arthritis/ or exp rheumatoid arthritis/ | 310664 |
| 19 | exp hip osteoarthritis/ or exp osteoarthritis/ | 72791 |
| 20 | (arthrit* or osteoarthrit* or osteoarthroses or "rheumatoid arthrit*").tw. | 189738 |
| 21 | 18 or 19 or 20 | 338180 |
| 22 | 17 and 21 | 10696 |
| 23 | meta analysis/ | 66936 |
| 24 | randomized controlled trial/ | 334512 |
| 25 | (metaanalys* or "meta analys*" or "meta-analys*").tw. | 60497 |
| 26 | (random* or "controlled trial*" or "clinical trial*" or rct).tw. | 968002 |
| 27 | "systematic review*".tw. | 47500 |
| 28 | 23 or 24 or 25 or 26 or 27 | 1114673 |
| 29 | 22 and 28 | 837 |
| 30 | limit 29 to (english language and yr="2002 -Current") | 645 |

**Appendix 2:**

**Figure S1.**

**Risk of bias graph for randomized controlled trials: review author’s judgments about each risk of bias item**

NA=not applicable; ITT=intention-to-treat; PP=per protocol

**Appendix 3:**

**Table S1.**

**Study and participant characteristics (randomized controlled trials)**

| **Author year**  **Ref ID**  **Country** | **Study details** | **Inclusion/ exclusion criteria** | **Intervention and prostheses used** | **Patient characteristics** | | | | | |
| --- | --- | --- | --- | --- | --- | --- | --- | --- | --- |
|  |  |  |  |  | | | **THR 1** | | **THR 2** |
| Angadi 2012^24^  UK | ***Comparison category :*** Cup fixation  ***Aim:*** To compare the clinical and radiological results of a cemented all-polyethylene Ultima acetabular component with those of a cementless porous-coated acetabular component (PFC) following total hip replacement (THR)  ***Length of follow up:*** up to 14 years [The mean follow-up was 7.52 years (0.4 to 15.0) for patients in Ultima group and 7.87 years (0.5 to 14.0) for those in PFC group] | ***Inclusion criteria:*** Candidates for primary THR with osteoarthritis or rheumatoid arthritis who were independently mobile without cognitive impairment  ***Exclusion criteria:*** Patients aged < 55 years of age, and those judged to be unsuitable for cementless fixation at surgery at the discretion of the senior operating surgeon, were excluded. Patients with cognitive impairment were excluded | ***THR 1:*** Cemented all-polyethylene Ultima acetabular component. The Ultima acetabular component is UHMWPE implant with a minimum thickness of 5 mm. It is hemispherical with a circumferential flange  ***THR 2:*** Cementless porous-coated acetabular component with a polyethylene liner. The PFC acetabular component is a cobalt-chromium alloy hemispherical shell without holes, which is a porous-coated surface of cobalt–chromium–molybdenum alloy beads with a mean pore size of 290 μm and an UHMWPE liner | ***Patients randomised*** | | | 183 | | 104 |
|  |  |  |  | ***Age (years) - Mean (SD/range)*** | | | 71.3 (55 – 89) | | 69.8 (56 – 89) |
|  |  |  |  | ***Sex - female n/N (%)*** | | | 110/183 (60) | | 56/104 (54) |
|  |  |  |  | ***Weight (kg) - Mean (SD/range)*** | | | 73.9 (43 – 128) | | 76.1 (49 – 124) |
|  |  |  |  | ***BMI (kg/m^2^) - Mean (SD/range)*** | | | 26.7 (13.3 – 41.4) | | 27.4 (18.8 – 44.1) |
|  |  |  |  | ***Primary osteoarthritis n/N (%)*** | | | 172/183 (94) | | 99/104 (95) |
|  |  |  |  | ***Bilateral osteoarthritis n/N (%)*** | | | 55/183 (30) | | 37/104 (36) |
|  |  |  |  | ***Harris Hip Score - Mean (SD/range)*** | | | 35.2 (9 – 76) | | 35.7 (10 – 70) |
|  |  |  |  | ***Oxford Hip Score - Mean (SD/range)*** | | | NR | | NR |
| Bjorgul 2010^67^ Bjorgul 2010^23^  Norway | ***Comparison category:*** Cup fixation  ***Aim:*** To compare the effects of cemented (Charnley) and uncemented (Duraloc) cups on long term follow up for radiographic and clinical outcomes  ***Length of follow up:***  10-14 years | ***Inclusion criteria:*** Patients aged 75 years or younger with OA, posttraumatic arthritis, psoriatic arthritis, gout, rheumatoid arthritis (RA), juvenile rheumatoid arthritis, and systemic lupus erythematosus  ***Exclusion criteria:*** Previous prosthetic replacement was a contraindication to participation, but not osteotomies and internal fixations | ***THR 1:*** THR using cemented (Charnley) cup  Cement containing gentamycin and a Charnley stem (DePuy) with 22.225 mm head diameter  ***THR 2:*** THR using uncemented (Duraloc) cup. Duraloc 1200 cup (DePuy); hemispherical modular cup consisting of a titanium shell with a porous-coated surface | ***Patients randomised*** | | | 107 | | 108 |
|  |  |  |  | ***Age (years) - Mean (SD/range)*** | | | 65 (95% CI 64, 66) | | 66 (95% CI 65, 67) |
|  |  |  |  | ***Sex - female n/N (%)*** | | | 81/107 (76.0) | | 76/108 (71.0) |
|  |  |  |  | ***Weight (kg) - Mean (SD/range)*** | | | NR | | NR |
|  |  |  |  | ***BMI (kg/m^2^) - Mean (SD/range)*** | | | 27 (95% CI 27, 28) | | 27 (95% CI 26, 27) |
|  |  |  |  | ***Primary osteoarthritis n/N (%)*** | | | 93/107 (87.0) | | 94/108 (87.0) |
|  |  |  |  | ***Bilateral osteoarthritis n/N (%)*** | | | 13/107 (12.1) | | 12/108 (11.1) |
|  |  |  |  | ***Harris Hip Score - Mean (SD/range)*** | | | 47 (95% CI 45, 50) | | 49 (95% CI 47, 52) |
|  |  |  |  | ***Oxford Hip Score - Mean (SD/range)*** | | | NR | | NR |
| McCalden 2009^68^  Canada | ***Comparison category:*** Cup liner bearing surface  ***Aim:*** To report the clinical and radiographic results, after a minimum of five years of follow-up, of a randomized, blinded, controlled trial comparing a conventional polyethylene (PE) with a first-generation highly cross-linked PE  ***Length of follow up:*** mean of 6.8 years | ***Inclusion criteria:*** A patient had to have degenerative arthritis of one hip requiring total hip arthroplasty, a designation of A or B according to the Charnley hip classification, and an age between 40 and 79  years  ***Exclusion criteria:*** Pre-existing bone disease (such as severe osteoporosis or osteomalacia), systemic conditions affecting bone density (such as inflammatory arthritis or renal disease), and a contralateral revision or poorly functioning THR | ***THR 1:*** Highly cross-linked PE acetabular cup liners. The highly cross-linked polyethylene liners, calcium-stearate-free GUR 1050 resin was also utilized to create compression-molded sheets, which were then machined into the final implant geometry  ***THR 2:*** Conventional PE acetabular cup liners. Conventional polyethylene liners used in this study were made of calciumstearate- free GUR 1050 resin machined from compressionmolded sheet polyethylene. The final implant was then sterilized with gamma radiation (25 kGy) in an inert nitrogen environment | ***Patients randomised*** | | | 50 | | 50 |
|  |  |  |  | ***Age (years) - Mean (SD/range)*** | | | 72.31 (56-79) | | 72.58 (56-79) |
|  |  |  |  | ***Sex - female n/N (%)*** | | | 33/50 (66%) | | 36/50 (72%) |
|  |  |  |  | ***Weight (kg) - Mean (SD/range)*** | | | NR | | NR |
|  |  |  |  | ***BMI (kg/m^2^) - Mean (SD/range)*** | | | 29.7 (22-39) | | 29.71 (18-48) |
|  |  |  |  | ***Primary osteoarthritis n/N (%)*** | | | NR | | NR |
|  |  |  |  | ***Bilateral osteoarthritis n/N (%)*** | | | NR | | NR |
|  |  |  |  | ***Harris Hip Score - Mean (SD/range)*** | | | 38.96 (11.35) | | 35.64 (12.97) |
|  |  |  |  | ***Oxford Hip Score - Mean (SD/range)*** | | | NR | | NR |
| Engh 2012^26^ Engh 2006^69^  US | ***Comparison category:*** Cup liner bearing surface  ***Aim:*** To compare the clinical outcome of THR patients randomized to either cross-linked or conventional non– cross-linked PE cup liners  ***Length of follow up:*** 10 years | ***Inclusion criteria:*** NR  ***Exclusion criteria:*** NR | ***THR 1:*** Cross-linked PE cup liners (Marathon). Patients were implanted a Duraloc 100 (DePuy) cup incorporating a 4-mm lateralized liner, The polyethylene liner was secured in the Duraloc shell by a peripheral locking ring that engaged a groove machined into the liner and shell  ***THR 2:*** Non-cross linked (conventional) PE cup liners (Enduron) | ***Patients randomised*** | | | 111 | | 109 |
|  |  |  |  | ***Age (years) - Mean (SD/range)*** | | | 62.5 (10.6) | | 62.0 (11.1) |
|  |  |  |  | ***Sex - female n/N (%)*** | | | 65/111 (58.0) | | 57/109 (52.3) |
|  |  |  |  | ***Weight (kg) - Mean (SD/range)*** | | | 84.3 (21.3) | | 81.6 (18.1) |
|  |  |  |  | ***BMI (kg/m^2^) - Mean (SD/range)*** | | | 28.6 (5.5) | | 27.9 (5.1) |
|  |  |  |  | ***Primary osteoarthritis n/N (%)*** | | | 99/111 (89.1) | | 90/109 (82.5) |
|  |  |  |  | ***Bilateral osteoarthritis n/N (%)*** | | | NR | | NR |
|  |  |  |  | ***Harris Hip Score* - Mean (SD/range)*** | | | 88.0 (14.0) | | 86.0 (15.0) |
|  |  |  |  | ***Oxford Hip Score - Mean (SD/range)*** | | | NR | | NR |
| Capello 2008^28^ D’Antonio 2005^70^ D’Antonio 2003^30^ Mesko 2011^31^  US | ***Comparison category:*** Cup shell design  ***Aim:*** To compare clinical and radiography outcomes between patients receiving THR with ceramic-on-ceramic bearings vs. metal-on-polyethylene bearings; to compare the results between two groups of patients receiving THRs with ceramic-on-ceramic bearings but with different cup design (porous-coated shell vs. arc-deposited HA-coated shell)  ***Length of follow up:*** 10 years | ***Inclusion criteria:*** patients aged 21-75 years, not morbidly obese, clinically qualified for THR, diagnosis of osteoarthritis, traumatic arthritis, avascular necrosis, slipped capital epiphysis, pelvic fracture, femoral fracture, failed fracture, fixation, or diastrophic variant, absence of active infection in the affected hip or no previous THA, no psychiatric disorder, senile dementia, Alzheimer’s disease, presence of alcohol, or substance abuse, no neuromuscular or neurosensory deficiency, no systemic disorder, was not immunologically suppressed, nor was receiving steroids in excess of physiologic dose requirements; was skeletally mature, was not pregnant; and had no plans to relocate to another geographic area before completion of the study  ***Exclusion criteria:*** See above | ***THR 1:*** THR with ceramic-on-ceramic bearings (titanium porous-coated shell) – System I  ***THR 2:*** THR with ceramic-on-ceramic bearings (titanium arc-deposited HA-coated shell) – System II  ***THR 3:*** THR with metal-on-polyethylene bearings (titanium porous-coated shell) – System III  ***THR 4:*** This arm was added later and was not randomised arm; therefore not extracted – System IV | ***Patients randomised*** | | | ***THR 1:*** | ***THR 2:*** | ***THR 3:*** |
|  |  |  |  |  |  |  | 113 | 109 | 106 |
|  |  |  |  | ***Age (years) - Mean (SD/range)*** | | | 53 (11.4) | 54 (10.7) | 55 (10.7) |
|  |  |  |  | ***Sex - female n/N (%)*** | | | 41/113 (35.0) | 41/109 (37.0) | 42/106 (39.0) |
|  |  |  |  | ***Weight (kg) - Mean (SD/range)*** | | | 85.0 (17.9) | 87.8 (17.3) | 85.9 (17.7) |
|  |  |  |  | ***BMI (kg/m^2^) - Mean (SD/range)*** | | | NR | NR | NR |
|  |  |  |  | ***Primary osteoarthritis n/N (%)*** | | | 95/113 (84.0) | 86/109 (79.0) | 81/106 (77.0) |
|  |  |  |  | ***Bilateral osteoarthritis n/N (%)*** | | | NR | NR | NR |
|  |  |  |  | ***Harris Hip Score - Mean (SD/range)*** | | | NR | NR | NR |
|  |  |  |  | ***Oxford Hip Score - Mean (SD/range)*** | | | NR | NR | NR |
| Corten 2011^71^ Laupacis 2002^33^ Bourne 2010^72^  Corten 2011^73^  Canada | ***Comparison category:*** Cup/stem fixation and Femoral head bearing-on-cup liner bearing  ***Aim:*** To compare the effects of cemented cup/stem (Mallory-head) and uncemented cup/stem (Mallory-head) prosthesis on long term follow up for mortality, revision, time to revision, health –related quality of life, and radiography signs  ***Length of follow up:*** 20 years | ***Inclusion criteria:*** Osteoarthritis of the hip and undergoing a unilateral primary arthroplasty  ***Exclusion criteria:*** Patient’s age > 75 years, severe, symptomatic osteoarthritis of either knee or the contralateral hip, a previous arthroplasty of the ipsilateral hip, arthroplasty on the contralateral side more than five years before the most recent arthroplasty, or had had infectious arthritis | ***THR 1:*** THR using cemented femoral and cemented acetabular components. All patients were operated on by either surgeon with use of an identical direct lateral approach and within a vertical laminar airflow enclosure in which the surgical team wore bodyexhaust suits  ***THR 2:*** THR using uncemented femoral and uncemented acetabular components. The prosthesis (Mallory-Head; Biomet, Warsaw, Indiana), was made from a titanium alloy. The implant was a tapered design with a 3° taper and the proximal one-third was plasma-sprayed | ***Patients randomised*** | | | 124 | | 126 |
|  |  |  |  | ***Age (years) - Mean (SD/range)*** | | | 64 (8.0) | | 64 (7.0) |
|  |  |  |  | ***Sex - female n/N (%)*** | | | 60/124 (48) | | 60/126 (46) |
|  |  |  |  | ***Weight (kg) - Mean (SD/range)*** | | | NR | | NR |
|  |  |  |  | ***BMI (kg/m^2^) - Mean (SD/range)*** | | | NR | | NR |
|  |  |  |  | ***Primary osteoarthritis n/N (%)*** | | | NR | | NR |
|  |  |  |  | ***Bilateral osteoarthritis n/N (%)*** | | | 0/124 (0.0) | | 0/126 (0.0) |
|  |  |  |  | ***Harris Hip Score - Mean (SD/range)*** | | | 44 (11.0) | | 43 (10.0) |
|  |  |  |  | ***Oxford Hip Score - Mean (SD/range)*** | | | NR | | NR |
| Howie 2012^74^  Australia | ***Comparison category:*** Femoral head size  ***Aim:*** To compare the incidence of dislocation at one year after total hip arthroplasty between two groups of patients who had received 36-mm and 28-mm femoral head articulations  ***Length of follow up:*** 1 year | ***Inclusion criteria:*** Patients aged 60 years or older with osteoarthritis and rheumatoid arthritis referred for THR  ***Exclusion criteria:*** Patients younger than 60 years with diagnoses other than osteoarthritis, rheumatoid arthritis, inflammatory arthritis, or previous fracture/dislocation/surgery involving the hip, abnormal acetabulum, neuromuscular disorder affecting hip, tumour of the hip, unable to provide consent, unable to complete follow-up | ***THR 1:*** 36 mm femoral head***.*** All arthroplasties were performed with use of uncemented acetabular components, which comprised a cluster three-holed acetabular shell (Trilogy) fixed with one or two screws and a 10° elevated 36 or 28-mm inner-diameter highly cross-linked polyethylene liner (Longevity). A cemented femoral stem was used for all arthroplasties (CPT). During the trial, the  taper of the CPT femoral stem was changed from a 6° taper to a 12/14 taper by the manufacturer  ***THR 2:*** 28 mm femoral head. See above | ***Patients randomised*** | | | 273 | | 284 |
|  |  |  |  | ***Age (years) - Mean (SD/range)*** | | | 72.3 (95% CI: 71.5, 73.0) | | 72.3 (95% CI: 71.6, 73.1) |
|  |  |  |  | ***Sex - female n/N (%)*** | | | 152/273 (56.0) | | 175/284 (61.3) |
|  |  |  |  | ***Weight (kg) - Mean (SD/range)*** | | | NR | | NR |
|  |  |  |  | ***BMI (kg/m^2^) - Mean (SD/range)*** | | | 28.0 (95% CI: 27.4, 28.7) | | 28.4 (95% CI: 27.8, 29.0) |
|  |  |  |  | ***Primary osteoarthritis n/N (%)*** | | | 96.3 (95% CI: 94.1, 98.6) | | 95.4 (95% CI: 93.0, 97.9) |
|  |  |  |  | ***Bilateral osteoarthritis n/N (%)*** | | | 0 | | 0 |
|  |  |  |  | ***Harris Hip Score - Mean (SD/range)*** | | | NR | | NR |
|  |  |  |  | ***Oxford Hip Score - Mean (SD/range)*** | | | NR | | NR |
| Lewis 2008^75^  Canada | ***Comparison category:*** Femoral head bearing  ***Aim:*** To compare clinical outcomes in patients who received total hip replacement (THR) with oxinium vs. Cobalt chrome femoral heads  ***Length of follow up:*** 2 years | ***Inclusion criteria:*** NR  ***Exclusion criteria:*** NR | ***THR 1:*** THR with Oxinium femoral heads. 46 patients received an Echelon stem, the remaining 4 received a Synergy stem. The acetabular components were press fit, uncemented Reflection cups paired with either standard polyethylene (22 cases) or highly crosslinked polyethylene (28 cases)  ***THR 2:*** THR with Cobalt chrome femoral heads. Thirty patients received an Echelon stem whereas the remaining 20 patients received a Synergy stem. The acetabular components were press fit. Uncemented Reflection cups paired with eitlicr standard polyethylene (31 eases) or highly cross-linked polyethylene (19 cases) | ***Patients randomised*** | | | 50 | | 50 |
|  |  |  |  | ***Age (years) - Mean (SD/range)*** | | | 51 (10.8) | | 51 (11.0) |
|  |  |  |  | ***Sex - female n/N (%)*** | | | 24/50 (48.0) | | 24/50 (48.0) |
|  |  |  |  | ***Weight (kg) - Mean (SD/range)*** | | | NR | | NR |
|  |  |  |  | ***BMI (kg/m^2^) - Mean (SD/range)*** | | | NR | | NR |
|  |  |  |  | ***Primary osteoarthritis n/N (%)*** | | | NR | | NR |
|  |  |  |  | ***Bilateral osteoarthritis n/N (%)*** | | | NR | | NR |
|  |  |  |  | ***Harris Hip Score - Mean (SD/range)*** | | | NR | | NR |
|  |  |  |  | ***Oxford Hip Score - Mean (SD/range)*** | | | NR | | NR |
| Amanatullah 2011^76^  USA | ***Comparison category:*** Femoral head bearing-on-cup liner bearing  ***Aim:*** To compare the clinical performance and evaluate the wear rate of ceramic-on-ceramic vs. ceramic-on-polyethylene (PE) bearing surfaces  ***Length of follow up:*** >5 years | ***Inclusion criteria:*** Patients were included if clinically indicated for a THA as a result of osteoarthritis or rheumatoid arthritis and were 21 to 80 years of age with a Harris Hip Score <=60, availability for => 2 years of clinical follow-up, ability to meet acceptable preoperative medical clearance, and without the presence or history of treatment for cardiac, pulmonary, hematologic, or any other medical condition that would pose excessive operative risk  ***Exclusion criteria:*** NR | ***THR 1:*** Ceramic-on-ceramic. Each ceramic-ceramic articulation was implanted with a 28- or 32-mm alumina ceramic femoral head and an alumina ceramic acetabular cup liner  ***THR 2:*** Ceramic-on-polyethylene. Alumina ceramic components were also sterilized with ethylene oxide gas. Metal components were sterilized with a minimum of 25 kGy of gamma irradiation | ***Patients randomised*** | | | 166 | | 146 |
|  |  |  |  | ***Age (years) - Mean (SD/range)*** | | | 50.4 (12.8) | | 54.7 (12.9) |
|  |  |  |  | ***Sex - female n/N (%)*** | | | 60 (36.1) | | 62 (42.5) |
|  |  |  |  | ***Weight (kg) - Mean (SD/range)*** | | | 86.9 (20.0) | | 83.7 (18.5) |
|  |  |  |  | ***BMI (kg/m^2^) - Mean (SD/range)*** | | | 29.6 (12.4) | | 28.0 (5.1) |
|  |  |  |  | ***Primary osteoarthritis n/N (%)*** | | | NR | | NR |
|  |  |  |  | ***Bilateral osteoarthritis n/N (%)*** | | | NR | | NR |
|  |  |  |  | ***Harris Hip Score - Mean (SD/range)*** | | | NR | | NR |
|  |  |  |  | ***Oxford Hip Score - Mean (SD/range)*** | | | NR | | NR |
| Kadar 2011^39^  Norway | ***Comparison category:*** Femoral head bearing-on-cup liner bearing  ***Aim:*** To evaluate wear and migration patterns between cemented highly cross-linked Reflection All-Poly (XLPE) cup and All-Poly cup articulated with either Oxinium or cobalt chrome (CoCr) femoral heads compared to the Charnley Ogee prostheses  ***Length of follow up:*** 2 years | ***Inclusion criteria:*** Primary or secondary osteoarthritis of the hip  ***Exclusion criteria:*** BMI > 35, incompensated cardio-pulmonary disease, malignant disease, dementia, rheumatoid arthritis,  or other serious systemic diseases | ***THR 1:*** Charnley Ogee prostheses. Charnley monoblock stainless steel femoral stem with  a 22.2 mm head articulated with a cemented Charnley  Ogee UHMWPE (GUR 1050) acetabular cup that was g-sterilized with 2.5 Mrad in nitrogen  ***THR 2:*** Cobalt chrome-on-polyethylene (PE) articulation. Spectron EF femoral stem with a 28 mm CoCr femoral head and a Reflection All-Poly UHMWPE (GUR 1050) cup that was sterilized by EtO  ***THR 3:*** Oxinium-on-polyethylene (PE) articulation***.*** Spectron EF femoral stem with a 28 mm Oxinium femoral head and a Reflection All-Poly UHMWPE (GUR 1050) cup that was sterilized by EtO  ***THR 4:*** Cobalt chrome-on-HXLPE articulation. Spectron EF femoral stem with a 28 mm CoCr femoral head and a Reflection All-Poly XLPE (GUR 1050) cup irradiated with 10 Mrad, melted at 1358C, and EtO  sterilized  ***THR 5:*** Oxinium-on-HXLPE articulation. Spectron EF femoral stem with a 28 mm Oxinium femoral head and a Reflection All-Poly XLPE (GUR 1050) cup irradiated with 10 Mrad, melted at 1358C, and EtO sterilized | ***Patients randomised*** | ***THR 1:*** | ***THR 2:*** | ***THR 3:*** | ***THR 4:*** | ***THR***  ***5:*** |
|  |  |  |  |  | 30 | 30 | 30 | 30 | 30 |
|  |  |  |  | ***Age (years) - Mean (SD/range)*** | 70 (6.1) | 69 (5.9) | 69 (6.7) | 70 (5.3) | 70 (5.4) |
|  |  |  |  | ***Sex - female n/N (%)*** | 20/30 (66.6) | 20/30 (66.6) | 23/30 (76.6) | 20/30 (66.6) | 22/30 (73.3) |
|  |  |  |  | ***Weight (kg) - Mean (SD/range)*** | 76 (14.9) | 76 (11.1) | 72 (13.9) | 80 (14.8) | 76 (14.6) |
|  |  |  |  | ***BMI (kg/m^2^) - Mean (SD/range)*** | NR | NR | NR | NR | NR |
|  |  |  |  | ***Primary osteoarthritis n/N (%)*** | 28/30 (93.3) | 26/30 (86.6) | 26/30 (86.6) | 22/30 (73.3) | 27/30 (90.0) |
|  |  |  |  | ***Bilateral osteoarthritis n/N (%)*** | NR | NR | NR | NR | NR |
|  |  |  |  | ***Harris Hip Score - Mean (SD/range)*** | 45 (NR) | 41 (NR) | 47 (NR) | 47 (NR) | 40 (NR) |
|  |  |  |  | ***Oxford Hip Score - Mean (SD/range)*** | NR | NR | NR | NR | NR |
| Healy 2009^77^  US | ***Comparison category:***  Stem composition  ***Aim:*** To compare the effects of Cobalt chrome vs. Titanium femoral stems in terms of post-total hip replacement (THP) clinical and radiographic measures  ***Length of follow up:*** 4.7 (2.0-8.9) years | ***Inclusion criteria:*** NR  ***Exclusion criteria:*** NR | ***THR 1:*** THR with cobalt chromium femoral stem. Trilock femoral stem made of cobalt-chrome or titanium in 11 sizes is a straight, collarless, modular, cementless, porous-coated femoral implant with a flat, tapered, wedge design of the intraosseous body of the stem  ***THR 2:*** THR with titanium femoral stem. See full data extraction sheet for more details | ***Patients randomised*** | | | 199 | | 191 |
|  |  |  |  | ***Age (years) - Mean (SD/range)*** | | | 66 (25-100) | | 64 (35-102) |
|  |  |  |  | ***Sex - female n/N (%)*** | | | 96/199 (48.2) | | 92/191 (48.1) |
|  |  |  |  | ***Weight (kg) - Mean (SD/range)*** | | | 81.7 (44.9-136.0) | | 81.7 (36.3-149.6) |
|  |  |  |  | ***BMI (kg/m^2^) - Mean (SD/range)*** | | | NR | | NR |
|  |  |  |  | ***Primary osteoarthritis n/N (%)*** | | | 182/199 (91.4) | | 168/191 (88.0) |
|  |  |  |  | ***Bilateral osteoarthritis n/N (%)*** | | | 33/199 (16.6) | | 24/191 (12.6) |
|  |  |  |  | ***Harris Hip Score - Mean (SD/range)*** | | | 50.3 (16.2-70.7) | | 50.8 (23.4-75.4) |
|  |  |  |  | ***Oxford Hip Score - Mean (SD/range)*** | | | NR | | NR |
| Kim 2011^78^  South Korea | ***Comparison category:***  Stem design  ***Aim:*** Comparing a short metaphyseal-fitting femoral stem to conventional metaphyseal- and diaphyseal-filling stem with respect to post-operative clinical and radiographic parameters  ***Length of follow up:*** 3.35 years | ***Inclusion criteria:*** NR  ***Exclusion criteria:*** Severe osteoporosis of the proximal femur | ***THR 1:*** Short anatomical metaphyseal-fitting cementless stem (Proxima). Cementless Pinnacle acetabular component made of titanium alloy, Proxima stem, and 28-mm-internal-diameter Biolox forte ceramic liner were used in all hips  ***THR 2:*** Conventional anatomical metaphyseal- and diaphyseal-filling cementless stem (Profile). The cementless Profile femoral component is made of titanium alloy and is an anatomical metaphyseal- and diaphyseal-fitting stem | ***Patients randomised*** | | | 50 | | 50 |
|  |  |  |  | ***Age (years) - Mean (SD/range)*** | | | 54.3 (12.97) | | 51.8 (12.3) |
|  |  |  |  | ***Sex - female n/N (%)*** | | | 28/50 (56) | | 26/50 (52) |
|  |  |  |  | ***Weight (kg) - Mean (SD/range)*** | | | 66.5 (9.51) | | 64.8 (10.6) |
|  |  |  |  | ***BMI (kg/m^2^) - Mean (SD/range)*** | | | 25.6 (2.82) | | 24.7 (3.6) |
|  |  |  |  | ***Primary osteoarthritis n/N (%)*** | | | 24/50 (48) | | 24/50 (48) |
|  |  |  |  | ***Bilateral osteoarthritis n/N (%)*** | | | 5/50 (10) | | 5/50 (10) |
|  |  |  |  | ***Harris Hip Score - Mean (SD/range)*** | | | 44.6 (NR) | | 48.4 (NR) |
|  |  |  |  | ***Oxford Hip Score - Mean (SD/range)*** | | | NR | | NR |
| Kim 2011^79^  South Korea | ***Comparison category:***  Stem fixation  ***Aim:*** To compare the clinical and radiological results, rates of revision, and the survival of implants after THRs with cemented (hybrid) vs. cementless femoral components performed in patients < 50 years of age at a minimum 16 years’ follow-up  ***Length of follow up:*** 20 years | ***Inclusion criteria:*** NR  ***Exclusion criteria:*** NR | ***THR 1:*** THR with cemented femoral stem. The Charnley Elite or Elite-plus stem (Ortron 90) (DePuy, Leeds, United Kingdom) was used in the cemented (hybrid) group and the Profile stem (DePuy) in the cementless group  ***THR 2:*** THR with cementless femoral stem. A cementless Duraloc 100 or 1200 series acetabular component (DePuy, Warsaw, Indiana) was used in all hips in both groups. Of the 62 Duraloc 1200 acetabular components used in both groups, 28 were fixed with one or two screws and the remaining 34 were press-fitted without using an additional screw | ***Patients randomised*** | | | 83 | | 83 |
|  |  |  |  | ***Age (years) - Mean (SD/range)*** | | | 43.4 (21 - 50) | | 46.8 (21 - 49) |
|  |  |  |  | ***Sex - female n/N (%)*** | | | 16/78 (21) | | 21/79 (27) |
|  |  |  |  | ***Weight (kg) - Mean (SD/range)*** | | | 59 (45 - 82) | | 60.5 (48 - 87) |
|  |  |  |  | ***BMI (kg/m^2^) - Mean (SD/range)*** | | | 22.2 (22.1 - 24.8) | | 22.2 (21.9 - 24.4) |
|  |  |  |  | ***Primary osteoarthritis n/N (%)*** | | | 10 (12.8) | | 12 (15.2) |
|  |  |  |  | ***Bilateral osteoarthritis n/N (%)*** | | | NR | | NR |
|  |  |  |  | ***Harris Hip Score - Mean (SD/range)*** | | | 44 (5 - 66) | | 48.8 (6 - 55) |
|  |  |  |  | ***Oxford Hip Score - Mean (SD/range)*** | | | NR | | NR |
| Bascarevic 2010 ^43^ | ***Comparison category:***  Femoral head bearing-on-cup liner bearing  ***Aim:*** To compare the clinical and radiological results, rates of revision, and complications between THRs with ceramic-on-ceramic vs. metal-on-cross-linked polyethylene bearings in patients < 65 years of age at 4 years’ follow-up  ***Length of follow up:*** 5 years | ***Inclusion criteria:*** Hip OA, age <65 years, and high activity level  ***Exclusion criteria:*** history of hip infection, degenerative damage of the joint due to infection | Zimmer stem VerSys porous collared, fibre metal midcoat; acetabular ring Trilogy, fibre metal-coated;  the external geometry of the metal components  was identical regardless of the bearing couple  ***THR 1:*** THR with cluster-hole press fit shell, alumina ceramic acetabular insert, alumina ceramic femoral head, and porous collared stem  ***THR 2:*** THR with multi-hole press fit shell, cross-linked polyethylene acetabular insert, cobalt chrome femoral head, and porous collared stem | ***Patients randomised*** | | | 78 | | 72 |
|  |  |  |  | ***Age (years) - Mean (SD/range)*** | | | 54 (7.1) | | 55.6 (6.5) |
|  |  |  |  | ***Sex - female n/N (%)*** | | | 62/78 (79) | | 50/72 (69) |
|  |  |  |  | ***Weight (kg) - Mean (SD/range)*** | | | NR | | NR |
|  |  |  |  | ***BMI (kg/m2) - Mean (SD/range)*** | | | 26.7 (3.8) | | 27.8 (4.2) |
|  |  |  |  | ***Primary osteoarthritis n/N (%)*** | | | 44/78 (56) | | 41/72 (57) |
|  |  |  |  | ***Bilateral osteoarthritis n/N (%)*** | | | NR | | NR |
|  |  |  |  | ***Harris Hip Score - Mean (SD/range)*** | | | 45.6 (8.2) | | 43.1 (8.2) |
|  |  |  |  | ***Oxford Hip Score - Mean (SD/range)*** | | | NR | | NR |
| THR=total hip replacement; SD=standard deviation; BMI=body mass index; OA=osteoarthritis; NR=not reported; HXLPE=highly cross-linked polyethylene; HA=hydroxyapatite; UHMWPE=ultra-high molecular- weight polyethylene; CoCr=cobalt chrome. * Modified HHS | | | | | | | | | |

**Appendix 4:**

**Table S2.**

**Risk of bias summary table for randomized controlled trials: review author’s judgments about each risk of bias item**

| **First author, year, study ID** | **Selection bias**  Random sequence generation | **Selection bias**  Allocation concealment | **Performance bias**  Subjective  (e.g., patient-reported) | **Performance bias**  Objective  (e.g., mortality, radiography, dislocation) | **Detection bias**  Subjective  (e.g., patient-reported) | **Detection bias**  Objective  (e.g., mortality, radiography, dislocation) | **Attrition bias**  Subjective  (e.g., patient-reported) | **Attrition bias**  Objective  (e.g., mortality, radiography, dislocation) | **Reporting bias**  Selective reporting of the outcome, subgroups, or analysis | **Other bias**  Funding source, adequacy of statistical methods used, type of analysis [ITT/PP], baseline imbalance in important characteristics |
| --- | --- | --- | --- | --- | --- | --- | --- | --- | --- | --- |
| Angadi 2012^24^ |  |  |  |  |  |  |  |  |  |  |
| Bjorgul 2010^22^ |  |  |  |  |  |  |  |  |  |  |
| McCalden 2009^25^ |  |  |  |  |  |  |  |  |  |  |
| Engh 2012^26^ |  |  |  |  |  |  |  |  |  |  |
| Capello 2008^28^ |  |  |  |  |  |  |  |  |  |  |
| Corten 2011^32^ |  |  |  |  |  |  |  |  |  |  |
| Howie 2012^36^ |  |  | NA |  | NA |  | NA |  |  |  |
| Lewis 2008^37^ |  |  |  |  |  |  |  |  |  |  |
| Amanatullah 2011^38^ |  |  |  |  |  |  |  |  |  |  |
| Kadar 2011^39^ |  |  |  |  |  |  |  |  |  |  |
| Healy 2009^40^ |  |  |  |  |  |  |  |  |  |  |
| Kim 2011^41^ |  |  |  |  |  |  |  |  |  |  |
| Kim 2011^42^ |  |  |  |  |  |  |  |  |  |  |
| Bascarevic 2010^43^ |  |  |  |  |  |  |  |  |  |  |
| ID=identification; ITT=intention-to-treat; PP=per protocol | | | | | | | | | | |

**Appendix 5:**

**Table S3.**

*NB: Tables 3-18 (results for specific outcomes reported in randomized controlled trials)*

**Harris Hip score (range: 0-100)**

| **Follow-up** | **Arm-specific estimates**  **n/N or mean (SD or 95% CI)** | **Difference**  **(p value or 95% CI)** | **# of RCTs**  **[SROB across studies]**** | **Treatment effect**  **Conclusion*** |
| --- | --- | --- | --- | --- |
| **Cup fixation**  **Cemented vs. Cementless** | | | | |
| 6 mo  2 yrs  5 yrs  10 yrs  10 yrs | 90.2 (87.9, 92.6) vs. 89.1 (86.9, 91.3)^22^  92.7 (89.6, 95.8) vs. 94.0 (92.4, 95.7)^22^  93.9 (91.6, 96.2) vs. 91.4 (89.3, 93.5)^22^  89.8 (87.0, 92.6) vs. 87.3 (84.1, 90.6)^22^  74.5 (NR) vs. 78.0 (NR)^24^ | p>0.05 (NS)  p>0.05 (NS)  p>0.05 (NS)  p>0.05 (NS)  p>0.05 (NS) | 2 [unclear ROB] | No difference |
| **Cup liner bearing surface**  **XLPE vs. Non XLPE** | | | | |
| 1 yr  5 yrs  10 yrs | 85.0 ( 10.3) vs. 83.4 (13.1)^25^  86.0 (13.1) vs. 83.1 (15.4)^25^  88.0 (14.0) vs. 86.0 (15.0)^26^ | MD=1.60, 95% CI: -3.07, 6.27^£^  MD=2.90, 95% CI: -2.77, 8.57^£^  MD=2.00, 95% CI: -1.85, 5.85^£^  **Pooled estimate of MD**^£^  2.29, 95% CI: -0.88, 5.45^25;26^ | 2 [unclear ROB] | No difference |
| **Cup shell design**  **Porous-coated shell vs. Arc-deposited HA-coated shell** | | | | |
| 5 yrs  10 yrs | 97.0 (NR) vs. 96.4 (NR)^28^  96.0 (NR) vs. 96.7 (NR)^28^ | p>0.05 (NS)  p>0.05 (NS) | 1 [unclear ROB] | Inconclusive |
| **Cup and femoral stem fixation**  **Cemented cup/femoral stem vs. Cementless cup/femoral stem** | | | | |
| 3 mo  6 mo  1 yr  3 yrs  5 yrs  7 yrs | 41 (12.0) vs. 41 (11.0)^32^  47 (12) vs. 50 (13)^32^  52 (10.0) vs. 53 (11.0)^32^  50 (14.0) vs. 52 (11.0)^32^  47 (14.0) vs. 48 (13.0)^32^  44 (15) vs. 46 (14)^32^ | MD=0.0, 95% CI: -3.00, 3.00^£^  MD=-3.0, 95% CI: -6.32, 0.32^£^  MD=-1.0, 95% CI: -3.86, 1.86^£^  MD=-2.0, 95% CI: -5.62, 1.62^£^  MD=-1.0, 95% CI: -4.88, 2.87^£^  MD=-2.0, 95% CI: -7.07, 3.05^£^ | 1 [low ROB] | No difference |
| **Femoral head bearing surface**  **Oxinium femoral heads vs. CoCr femoral heads** | | | | |
| 2 yrs | 92 (NR) vs. 92.5 (NR)^37^ | p>0.159 (NS) | 1 [unclear ROB] | Inconclusive |
| **Femoral head-on-cup liner bearing surfaces-I**  **Ceramic-on-Ceramic vs. Metal-on-PE** | | | | |
| 5 yrs  10 yrs | 96.4 (NR) vs. 97.0 (NR)^28^  96.7 (NR) vs. 96.4 (NR)^28^ | p>0.05 (NS)  p>0.05 (NS) | 1 [unclear ROB] | Inconclusive |
| **Femoral head-on-cup liner bearing surfaces -II**  **Ceramic-on-Ceramic vs. Ceramic-on-PE** | | | | |
| 5 yrs | NR^38^ | p>0.05 (NS) | 1 [unclear ROB] | Inconclusive |
| **Femoral head-on-cup liner bearing surfaces–III**  **Steel-on-PE vs. CoCr-on-PE vs. Oxinium-on-PE vs. CoCr-on-XLPE vs. Oxinium-on-XLPE** | | | | |
| 2 yrs | 91 (10.8) vs. 91 (8.5) vs. 91 (11.1) vs. 93 (11.3) vs. 88 (9.5)^39^ | p=0.7 (NS)  ANOVA-based p=0.5 (NS)^£^ | 1 [low ROB] | No difference |
| **Femoral head-on-cup liner bearing surfaces–IV**  **Ceramic-on-Ceramic vs. CoCr-on-XLPE** | | | | |
| 4 yrs | 95.1 (3.9) vs. 93.8 (5.1)^43^ | MD=1.3, 95% CI: -0.15, 2.75^£^  p=0.07 (NS) | 1 [unclear ROB] | No difference |
| **Femoral stem composition**  **CoCr vs. Titanium** | | | | |
| 5 yrs | 83 (NR) vs. 87 (NR)^40^ | p=0.029 (SS) | 1 [high ROB] | Inconclusive |
| **Femoral stem design**  **Short metaphyseal-fitting stem vs. Conventional metaphyseal- and diaphyseal-filling stem** | | | | |
| 3 yrs | 97.0 (NR) vs. 96.0 (NR)^41^ | p=0.79 (NS) | 1 [unclear ROB] | Inconclusive |
| **Femoral stem fixation**  **Cemented vs. Cementless** | | | | |
| 18 yrs | 91 (NR) vs. 90 (NR)^42^ | p=0.71(NS) | 1 [unclear ROB] | Inconclusive |
| SROB=summary risk of bias; MD=mean difference; SD=standard deviation; 95% CI=95 percent confidence interval; NR=not reported; SS=statistically significant; NS=statistically not significant; mo=month(s); yr(s)=year(s); HXLPE=highly cross-linked polyethylene; XLPE= cross-linked polyethylene; PE=polyethylene; HA=hydroxyapatite; CoCr= cobalt chrome | | | | |

Only those comparisons listed for which any evidence for the given outcome was reported

* Favours THR-1 (or THR-2), no difference, or inconclusive

** Decision was consensus-based

£ Calculated

**Appendix 6:**

**Table S4.**

**The Western Ontario and McMaster University Osteoarthritis Index (range: 0-100)**

| **Follow-up** | **Arm-specific estimates**  **n/N or mean (SD or 95% CI)** | **Difference**  **(p value or 95% CI)** | **# of RCTs**  **[SROB across studies]**** | **Treatment effect**  **Conclusion*** |
| --- | --- | --- | --- | --- |
| **Cup liner bearing surface**  **XLPE vs. Non XLPE** | | | | |
| 1 yr  5 yrs | 83.0 (17.2) vs. 81.6 (17.6)^25^  78.0 ( 19.4) vs. 78.1 (18.2)^25^ | MD=1.43, 95% CI: -5.48, 8.34^£^  MD=-0.12, 95% CI: -7.58, 7.34^£^ | 1 [unclear ROB] | No difference |
| **Cup and femoral stem fixation**  **Cemented cup/femoral stem vs. Cementless cup/femoral stem** | | | | |
| NA | Mean domain sub-scores only^32^ | - | 1 [low ROB] | NA |
| **Femoral head bearing surface**  **Oxinium femoral heads vs. CoCr femoral heads** | | | | |
| 2 yrs | 84.9 (NR) vs. 87.0 (NR)^37^ | p>0.159 (NS) | 1 [unclear ROB] | Inconclusive |
| **Femoral stem fixation**  **Cemented vs. Cementless** | | | | |
| 16 yrs | 11 (NR) vs. 13 (NR)^42^ | p=0.927(NS) | 1 [unclear ROB] | Inconclusive |
| SROB=summary risk of bias; SD=standard deviation; 95% CI=95 percent confidence interval; NR=not reported; SS=statistically significant; NS=statistically not significant; mo=month(s); yr(s)=year(s); HXLPE=highly cross-linked polyethylene; XLPE= cross-linked polyethylene; PE=polyethylene; HA=hydroxyapatite; CoCr= cobalt chrome; MD=mean difference | | | | |

Only those comparisons listed for which any evidence for the given outcome was reported

* Favours THR-1 (or THR-2), no difference, or inconclusive

** Decision was consensus-based

£ Calculated

**Appendix 7:**

**Table S5.**

**The McMaster-Toronto Arthritis Patient Preference Disability Questionnaire score (range: 0-30)**

| **Follow-up** | **Arm-specific estimates**  **n/N or mean (SD or 95% CI)** | **Difference**  **(p value or 95% CI)** | **# of RCTs**  **[SROB across studies]**** | **Treatment effect**  **Conclusion*** |
| --- | --- | --- | --- | --- |
| **Cup and femoral stem fixation**  **Cemented cup/femoral stem vs. Cementless cup/femoral stem** | | | | |
| 3 mo  6 mo  1 yr  3 yrs  5 yrs  7 yrs | **Mean change (post-operative)**  -5.3 (2.5) vs. -5.2 (2.2)^32^  -6.6 (1.9) vs. -6.4 (2.1)^32^  -7.0 (1.8) vs. -6.9 (2.0)^32^  -6.6 (2.3) vs. -6.4 (2.3)^32^  -6.0 (2.8) vs. -6.2 (2.4)^32^  -6.2 (2.8) vs. -6.0 (2.6)^32^ | **Mean change difference**  MD=0.10, 95% CI: -0.51, 0.71^£^  MD=0.20, 95% CI: -0.33, 0.73^£^  MD=0.10, 95% CI: -0.41, 0.61^£^  MD=0.20, 95% CI: -0.46, 0.86^£^  MD=-0.20, 95% CI: -0.45, 0.55^£^  MD=0.20, 95% CI: -0.74, 1.14^£^ | 1 [low ROB] | No difference |
| SROB=summary risk of bias; MD=mean difference; SD=standard deviation; 95% CI=95 percent confidence interval; NR=not reported; SS=statistically significant; NS=statistically not significant; mo=month(s); yr(s)=year(s) | | | | |

Only those comparisons listed for which any evidence for the given outcome was reported

* Favours THR-1 (or THR-2), no difference, or inconclusive

** Decision was consensus-based

£ Calculated

**Appendix 8:**

**Table S6.**

**Merle D'Aubigne and Postel score (range: 0-18)**

| **Follow-up** | **Arm-specific estimates**  **n/N or mean (SD or 95% CI)** | **Difference**  **(p value or 95% CI)** | **# of RCTs**  **[SROB across studies]**** | **Treatment effect**  **Conclusion*** |
| --- | --- | --- | --- | --- |
| **Cup and femoral stem fixation**  **Cemented cup/femoral stem vs. Cementless cup/femoral stem** | | | | |
| 3 mo  6 mo  1 yr  3 yrs  5 yrs  7 yrs | **Mean change (post-operative)**  5.8 (1.9) vs. 5.6 (2.2)^32^  6.7 (2.1) vs. 7.0 (2.2)^32^  7.5 (1.8) vs. 7.4 (2.1)^32^  7.1 (2.2) vs. 6.9 (2.1)^32^  6.5 (2.3) vs. 6.6 (2.4)^32^  6.1 (2.6) vs. 6.5 (2.8)^32^ | **Mean change difference**  MD=0.20, 95% CI: -0.34, 0.74^£^  MD=-0.30, 95% CI: -0.87, 0.27^£^  MD=0.10, 95% CI: -0.43, 0.63^£^  MD=0.20, 95% CI: -0.41, 0.81^£^  MD=-0.10, 95% CI: -0.77, 0.57^£^  MD=-0.40, 95% CI: -1.34, 0.54^£^ | 1 [low ROB] | No difference |
| SROB=summary risk of bias; MD=mean difference; SD=standard deviation; 95% CI=95 percent confidence interval; NR=not reported; SS=statistically significant; NS=statistically not significant; mo=month(s); yr(s)=year(s) | | | | |

Only those comparisons listed for which any evidence for the given outcome was reported

* Favours THR-1 (or THR-2), no difference, or inconclusive

** Decision was consensus-based

£ Calculated

**Appendix 9:**

**Table S7.**

**The University of California, Los Angeles activity scale (range: 1-10)**

| **Follow-up** | **Arm-specific estimates**  **n/N or mean (SD or 95% CI)** | **Difference**  **(p value or 95% CI)** | **# of RCTs**  **[SROB across studies]**** | **Treatment effect**  **Conclusion*** |
| --- | --- | --- | --- | --- |
| **Femoral stem fixation**  **Cemented vs. Cementless** | | | | |
| 16 yrs | 7.6 (NR) vs. 7.8 (NR)^42^ | p=0.814 (NS) | 1 [unclear ROB] | Inconclusive |
| SROB=summary risk of bias; SD=standard deviation; 95% CI=95 percent confidence interval; NR=not reported; SS=statistically significant; NS=statistically not significant; mo=month(s); yr(s)=year(s); MD=mean difference | | | | |

Only those comparisons listed for which any evidence for the given outcome was reported

* Favours THR-1 (or THR-2), no difference, or inconclusive

** Decision was consensus-based

**Appendix 10:**

**Table S8.**

**Short Form Health Survey (SF-12; range: 0-100)**

| **Follow-up** | **Arm-specific estimates**  **n/N or mean (SD or 95% CI)** | **Difference**  **(p value or 95% CI)** | **# of RCTs**  **[SROB across studies]**** | **Treatment effect**  **Conclusion*** |
| --- | --- | --- | --- | --- |
| **Cup liner bearing surface**  **XLPE vs. Non XLPE** | | | | |
| 1 yr  5 yrs | **Mental component score**  55.79 (7.38) vs. 56.01 (8.55)^25^  **Physical component score**  42.20 (11.37) vs. 40.86 (11.11)^25^  **Mental component score**  55.24 (8.01) vs. 53.36 (10.13)^25^  **Physical component score**  37.24 (12.16) vs. 40.00 (11.78)^25^ | **Mental component score**  MD=-0.22, 95% CI: -3.38, 2.94^£^  **Physical component score**  MD=1.34, 95% CI: -3.12, 5.80^£^  **Mental component score**  MD=1.88, 95% CI: -1.74, 5.50^£^  **Physical component score**  MD=-2.76, 95% CI: -7.51, 1.99^£^ | 1 [unclear ROB] | No difference |
| **Femoral head bearing surface**  **Oxinium femoral heads vs. CoCr femoral heads** | | | | |
| 2 yrs | **Mental component score**  53.80 (NR) vs. 52.57 (NR)^37^  **Physical component score**  45.20 (NR) vs. 49.20 (NR)^37^ | **Mental component score**  p>0.05 (NS)  **Physical component score**  p>0.05 (NS) | 1 [unclear ROB] | Inconclusive |
| **Femoral head-on-cup liner bearing surfaces**  **Ceramic-on-Ceramic vs. Ceramic-on-PE** | | | | |
| 5 yrs | NR^38^ | p>0.05 (NS) | 1 [unclear ROB] | Inconclusive |
| SROB=summary risk of bias; MD=mean difference; SD=standard deviation; 95% CI=95 percent confidence interval; NR=not reported; SS=statistically significant; NS=statistically not significant; mo=month(s); yr(s)=year(s); HXLPE=highly cross-linked polyethylene; XLPE= cross-linked polyethylene; PE=polyethylene; CoCr= cobalt chrome | | | | |

Only those comparisons listed for which any evidence for the given outcome was reported

* Favours THR-1 (or THR-2), no difference, or inconclusive

** Decision was consensus-based

^£^ Calculated

**Appendix 11:**

**Table S9.**

**Risk of revision (n/N)**

| **Follow-up** | **Arm-specific estimates**  **n/N or mean (SD or 95% CI)** | **Difference**  **(p value or 95% CI)** | **# of RCTs**  **[SROB across studies]**** | **Treatment effect**  **Conclusion*** |
| --- | --- | --- | --- | --- |
| **Cup fixation**  **Cemented vs. Cementless** | | | | |
| 10 yrs | 17/183 vs. 11/104^24^ | p>0.05 (NS); RR=0.87, 95% CI: 0.42, 1.80^£^ | 1 [low ROB] | Inconclusive |
| **Cup liner bearing surface**  **XLPE vs. Non XLPE** | | | | |
| 10 yrs | 2/111 vs. 11/109^26^ | p<0.05 (SS); RR=0.18, 95% CI: 0.04, 0.78^£^ | 1 [unclear ROB] | In favour of XLPE cup liner |
| **Cup shell design**  **Porous-coated shell vs. Arc-deposited HA-coated shell** | | | | |
| 5 yrs  5-10 yrs | 2/113 vs. 4/109^28^  2/113 vs. 2/109^28^ | p>0.05 (NS); RR=0.48, 95% CI: 0.09, 2.57^£^  p>0.05 (NS); RR=0.96, 95% CI: 0.13, 6.72^£^ | 1 [low ROB] | Inconclusive |
| **Cup and femoral stem fixation**  **Cemented cup/femoral stem vs. Cementless cup/femoral stem** | | | | |
| 7 yrs | 13/124 vs. 6/126^32^ | p=0.11 (NS); RR=2.20, 95% CI: 0.86, 5.60^£^ | 1 [low ROB] | Inconclusive |
| **Femoral head size**  **36 mm vs. 28 mm** | | | | |
| 1 yr | 4/273 vs. 6/284^36^ | p=NR; RR= 0.69, 95% CI: 0.19, 2.43^£^ | 1 [low ROB] | Inconclusive |
| **Femoral head bearing surface**  **Oxinium femoral heads vs. CoCr femoral heads** | | | | |
| 2 yrs | 1/50 vs. 1/50^37^ | p=NR; RR= 1.00, 95% CI: 0.06, 15.50^£^ | 1 [low ROB] | Inconclusive |
| **Femoral head-on-cup liner bearing surfaces-I**  **Ceramic-on-Ceramic vs. Metal-on-PE** | | | | |
| 5 yrs  5-10 yrs | 6/222 vs. 8/106^28^  4/222 vs. 5/106^28^ | p=0.045 (SS); RR= 0.35, 95% CI: 0.12, 1.00^£^  p=0.08 (NS); RR= 0.38, 95% CI: 0.10, 1.39^£^ | 1 [low ROB] | Inconclusive |
| **Femoral head-on-cup liner bearing surfaces-II**  **Ceramic-on-Ceramic vs. Ceramic-on-PE** | | | | |
| 5 yrs | 11/196 vs. 3/161^38^ | p=0.06 (NS); RR= 3.01, 95% CI: 0.85, 10.61^£^ | 1 [low ROB] | Inconclusive |
| **Femoral head-on-cup liner bearing surfaces–III**  **Ceramic-on-Ceramic vs. CoCr-on-XLPE** | | | | |
| 4 yrs | 0/78 vs. 2/72^43^ | p=0.23 (NS); RR and 95% CI not estimated | 1 [low ROB] | Inconclusive |
| **Femoral stem composition**  **CoCr vs. Titanium** | | | | |
| 5 yrs | 2/199 vs. 0/191^40^ | p=0.16 (NS); RR and 95% CI not estimated | 1 [unclear ROB] | Inconclusive |
| **Femoral stem design**  **Short metaphyseal-fitting stem vs. Conventional metaphyseal- and diaphyseal-filling stem** | | | | |
| 3 yrs | 0/50 vs. 0/50^41^ | p=NR; RR and 95% CI not estimated | 1 [low ROB] | Inconclusive |
| **Femoral stem fixation**  **Cemented vs. Cementless** | | | | |
| 20 yrs | Acetabular:  14/109 vs. 18/110^42^  Femoral:  3/109 vs. 4/110^42^ | p=0.673 (NS); RR= 0.78, 95% CI: 0.41, 1.49^£^  p=0.912 (NS); RR= 0.75, 95% CI: 0.17, 3.30^£^ | 1 [low ROB] | Inconclusive |
| RR=risk ratio (relative risk); SROB=summary risk of bias; SD=standard deviation; 95% CI=95 percent confidence interval; NR=not reported; SS=statistically significant; NS=statistically not significant; mo=month(s); yr(s)=year(s); HXLPE=highly cross-linked polyethylene; XLPE= cross-linked polyethylene; PE=polyethylene; HA=hydroxyapatite; CoCr= cobalt chrome | | | | |

Only those comparisons listed for which any evidence for the given outcome was reported

* Favours THR-1 (or THR-2), no difference, or inconclusive

** Decision was consensus-based

^£^ Calculated

**Appendix 12:**

**Table S10.**

**Risk of mortality (n/N)**

| **Follow-up** | **Arm-specific estimates**  **n/N or mean (SD or 95% CI)** | **Difference**  **(p value or 95% CI)** | **# of RCTs**  **[SROB across studies]**** | **Treatment effect**  **Conclusion*** |
| --- | --- | --- | --- | --- |
| **Cup fixation**  **Cemented vs. Cementless** | | | | |
| 10 yrs | 12/107 vs. 14/108^22^ | p=NR; RR=0.86, 95% CI: 0.41, 1.78^£^ | 1 [low ROB] | Inconclusive |
| **Cup liner bearing surface**  **XLPE vs. Non XLPE** | | | | |
| 5 yrs  10 yrs | 7/50 vs. 2/50^25^  17 /111 vs. 15/109^26^ | p>0.05 (NS); RR=3.50, 95% CI: 0.76, 16.03^£^  p>0.05 (NS); RR=1.11, 95% CI: 0.58, 2.11^£^  **Pooled estimate of MH-RR**  RR=1.39, 95% CI: 0.78, 2.49^25;26^ | 2 [unclear ROB] | Inconclusive |
| **Cup and femoral stem fixation**  **Cemented cup/femoral stem vs. Cementless cup/femoral stem** | | | | |
| 7 yrs | 18/124 vs. 17/126^32^ | p=NR; 1.07, 95% CI: 0.58, 1.98^£^ | 1 [low ROB] | Inconclusive |
| **Femoral head size**  **36 mm vs. 28 mm** | | | | |
| 1 yr | 5/273 vs. 2/284^36^ | p=NR; RR=2.58, 95% CI: 0.53, 13.20^£^ | 1 [low ROB] | Inconclusive |
| **Femoral stem design**  **Short metaphyseal-fitting stem vs. Conventional metaphyseal- and diaphyseal-filling stem** | | | | |
| 3 yrs | 0/50 vs. 0/50^41^ | p=NR; RR and 95% CI not estimated | 1 [low ROB] | Inconclusive |
| RR=risk ratio (relative risk); SROB=summary risk of bias; SD=standard deviation; 95% CI=95 percent confidence interval; NR=not reported; SS=statistically significant; NS=statistically not significant; mo=month(s); yr(s)=year(s); HXLPE=highly cross-linked polyethylene; XLPE= cross-linked polyethylene; PE=polyethylene; HA=hydroxyapatite; CoCr= cobalt chrome; MH-RR=Mantel-Haenszel relative risk | | | | |

Only those comparisons listed for which any evidence for the given outcome was reported

* Favours THR-1 (or THR-2), no difference, or inconclusive

** Decision was consensus-based

^£^ Calculated

**Appendix 13:**

**Table S11.**

**Femoral head penetration rate (mm/year)**

| **Follow-up** | **Arm-specific estimates**  **n/N or mean (SD or 95% CI)** | **Difference**  **(p value or 95% CI)** | **# of RCTs**  **[SROB across studies]**** | **Treatment effect**  **Conclusion*** |
| --- | --- | --- | --- | --- |
| **Cup liner bearing surface**  **XLPE vs. Non XLPE** | | | | |
| 5 yrs  5 yrs  10 yrs | 0.003 (-0.024, 0.030) vs. 0.051 (0.029, 0.073)^25^  0.24 (0.42) vs. 1.26 (0.62)^26^  0.06 (0.05) vs. 0.22 (0.11)^26^ | p = 0.006 (SS)  p<0.001 (SS)  p<0.001 (SS) | 2 [unclear ROB] | In favour of XLPE |
| **Femoral head-on-cup liner bearing surfaces**  **Steel-on-PE vs. CoCr-on-PE vs. Oxinium-on-PE vs. CoCr-on-XLPE vs. Oxinium-on-XLPE** | | | | |
| 2 yrs | 0.19 (0.16, 0.23) vs. 0.40 (0.33, 0.46) vs. 0.44 (0.37, 0.51) vs. 0.19 (0.15, 0.23) vs. 0.18 (0.13, 0.22)^39^ | p<0.001 (SS; steel-PE, CoCr- XLPE, and Oxinium-XLPE vs. CoCr-PE and Oxinium-PE) | 1 [low ROB] | In favour of CoCr- XLPE, Oxinium-XLPE, and steel-PE |
| SROB=summary risk of bias; MD=mean difference; SD=standard deviation; 95% CI=95 percent confidence interval; NR=not reported; SS=statistically significant; NS=statistically not significant; mo=month(s); yr(s)=year(s); HXLPE=highly cross-linked polyethylene; XLPE= cross-linked polyethylene; PE=polyethylene; CoCr= cobalt chrome | | | | |

Only those comparisons listed for which any evidence for the given outcome was reported

* Favours THR-1 (or THR-2), no difference, or inconclusive

** Decision was consensus-based

**Appendix 14:**

**Table S12.**

**Implant survival rate (%)**

| **Follow-up** | **Arm-specific Kaplan-Meier survival rate estimates (SD or 95% CI)** | **Difference**  **(p value or 95% CI)** | **# of RCTs**  **[SROB across studies]**** | **Treatment effect**  **Conclusion*** |
| --- | --- | --- | --- | --- |
| **Cup fixation**  **Cemented vs. Cementless** | | | | |
| 10 yrs  10 yrs | NR (NR) vs. NR (NR)^22^  86.8% (78.4, 92.1) vs. 89.2% (78.3, 94.8)^24^ | p=0.09 (NS)  log-rank test p=0.938 (NS) | 2 [low ROB] | No difference |
| **Cup liner bearing surface**  **XLPE vs. Non XLPE** | | | | |
| 10 yrs | 98.1% (2.7) vs. 92.6% (5.3)^26^ | p=0.02 (SS) | 1 [unclear ROB] | Inconclusive |
| **Cup shell design**  **Porous-coated shell vs. Arc-deposited HA-coated shell** | | | | |
| 5 yrs  10 yrs | 94.1% (87.5, 100) vs. 94.1% (87.5, 100)^28^  95.9% (91.9, 97.9) vs. 95.9% (91.9, 97.9)^28^ | p>0.05 (NS)  p>0.05 (NS) | 1 [low ROB] | No difference |
| **Cup and femoral stem fixation**  **Cemented cup/femoral stem vs. Cementless cup/femoral stem** | | | | |
| 5 yrs  10 yrs  15 yrs  20 yrs | 97.0% (95.0, 99.0) vs. 100.0% (NR)^32^  83.0% (79.0, 87.0) vs. 94.0% (92.0, 96.0)^32^  66.0% (61.0, 71.0) vs. 0.80% (76.0, 84.0)^32^  48.0% (41.0, 55.0) vs. 69.0% (64.0, 74.0)^32^ | p=0.007 (SS) | 1 [low ROB] | In favour of cementless cup and femoral stem |
| **Femoral head bearing surface**  **Oxinium femoral heads vs. CoCr femoral heads** | | | | |
| 2 yrs | 98.0% (NR) vs. 98.0% (NR) ^37^ | NR | 1 [low ROB] | Inconclusive |
| **Femoral stem composition**  **CoCr vs. Titanium** | | | | |
| 5 yrs | 98.9% (NR) vs. 100% (NR)^40^ | p=0.169 (NS) | 1 [unclear ROB] | Inconclusive |
| **Femoral stem fixation**  **Cemented vs. Cementless** | | | | |
| 20 yrs | Acetabular: 87.0% (80.0, 93.0) vs. 84.0% (78.0, 92.0)^42^  Femoral: 97.0% (91.0, 100.0) vs. 96.0% (93.0, 100.0)^42^ | p=0.776 (NS)  p=0.794 (NS) | 1 [low ROB] | No difference |
| RR=risk ratio (relative risk); SROB=summary risk of bias; SD=standard deviation; 95% CI=95 percent confidence interval; NR=not reported; SS=statistically significant; NS=statistically not significant; mo=month(s); yr(s)=year(s); HXLPE=highly cross-linked polyethylene; XLPE= cross-linked polyethylene; PE=polyethylene; CoCr= cobalt chrome | | | | |

Only those comparisons listed for which any evidence for the given outcome was reported

* Favours THR-1 (or THR-2), no difference, or inconclusive

** Decision was consensus-based

**Appendix 15:**

**Table S13.**

**Risk of implant dislocation (n/N)**

| **Follow-up** | **Arm-specific estimates**  **n/N or mean (SD or 95% CI)** | **Difference**  **(p value or 95% CI)** | **# of RCTs**  **[SROB across studies]**** | **Treatment effect**  **Conclusion*** |
| --- | --- | --- | --- | --- |
| **Cup fixation**  **Cemented vs. Cementless** | | | | |
| 10 yrs | 4/107 vs. 10/108^22^  1/183 vs. 3/104^24^ | p>0.05 (NS); RR= 0.40, 95% CI: 0.13, 1.24^£^  p=NR; RR= 0.18, 95% CI: 0.02, 1.79^£^  **Pooled estimate of Peto OR**^£^  OR=0.34, 95% CI: 0.13, 0.89^22;24^ | 2 [low ROB] | In favour of cemented cup |
| **Cup shell design**  **Porous-coated shell vs. Arc-deposited HA-coated shell** | | | | |
| 10 yrs | 2/113 vs. 3/109^28^ | p=NR; RR= 0.64, 95% CI: 0.10, 3.77^£^ | 1 [low ROB] | Inconclusive |
| **Femoral head size**  **36 mm vs. 28 mm** | | | | |
| 1 yr | 2/258 vs. 12/275^36^ | p=NR; RR= 0.17, 95% CI: 0.04, 0.78^£^ | 1 [low ROB] | In favour of 36 mm head size |
| **Femoral head bearing surface**  **Oxinium femoral heads vs. CoCr femoral heads** | | | | |
| 2 yrs | 2/50 vs. 1/50^37^ | p=NR; RR= 2.00, 95% CI: 0.18, 21.35^£^ | 1 [low ROB] | Inconclusive |
| **Femoral head-on-cup liner bearing surfaces -I**  **Ceramic-on-Ceramic vs. Ceramic-on-PE** | | | | |
| 5 yrs | 10/166 vs. 9/146^38^ | p=0.672 (NS); RR= 0.97, 95% CI: 0.40, 2.33^£^ | 1 [low ROB] | Inconclusive |
| **Femoral head-on-cup liner bearing surfaces-II**  **Ceramic-on-Ceramic vs. Metal-on-PE** | | | | |
| 10 yrs | 5/222 vs. 5/106^28^ | p=0.25 (NS); RR=0.47, 95% CI: 0.14, 1.61^£^ | 1 [low ROB] | Inconclusive |
| **Femoral head-on-cup liner bearing surfaces–III**  **Ceramic-on-Ceramic vs. CoCr-on-XLPE** | | | | |
| 4 yrs | 1/78 vs. 2/72^43^ | p=0.61 (NS); RR=0.46, 95% CI: 0.04, 5.06^£^ | 1 [low ROB] | Inconclusive |
| **Femoral stem composition**  **CoCr vs. Titanium** | | | | |
| 5 yrs | 3/199 vs. 0/191^40^ | p=0.678 (NS); RR and 95% CI not estimated | 1 [unclear ROB] | Inconclusive |
| RR=risk ratio (relative risk); OR=odds ratio; SROB=summary risk of bias; SD=standard deviation; 95% CI=95 percent confidence interval; NR=not reported; SS=statistically significant; NS=statistically not significant; mo=month(s); yr(s)=year(s); PE=polyethylene; HA=hydroxyapatite; CoCr= cobalt chrome | | | | |

Only those comparisons listed for which any evidence for the given outcome was reported

* Favours THR-1 (or THR-2), no difference, or inconclusive

** Decision was consensus-based

^£^ Calculated

**Appendix 16:**

**Table S14.**

**Risk of osteolysis (n/N)**

| **Follow-up** | **Arm-specific estimates**  **n/N or mean (SD or 95% CI)** | **Difference**  **(p value or 95% CI)** | **# of RCTs**  **[SROB across studies]**** | **Treatment effect**  **Conclusion*** |
| --- | --- | --- | --- | --- |
| **Cup fixation**  **Cemented vs. Cementless** | | | | |
| 10 yrs | 0/183 vs. 1/104^24^ | p=NR; RR and 95% CI not estimated | 1 [low ROB] | Inconclusive |
| **Cup liner bearing service**  **XLPE vs. Non XLPE** | | | | |
| 5 yrs  10 yrs | 0/50 vs. 0/50^25^  0/111 vs. 15/109^26^ | p=NA; RR and 95% CI not estimated  p<0.001; RR and 95% CI not estimated | 2 [unclear ROB] | Inconclusive |
| **Cup shell design**  **Porous-coated shell vs. Arc-deposited HA-coated shell** | | | | |
| 10 yrs | 1/113 vs. 2/109^28^ | p=NR; RR= 0.48, 95% CI: 0.04, 5.24^£^ | 1 [low ROB] | Inconclusive |
| **Femoral head-on-cup liner bearing surfaces -I**  **Ceramic-on-Ceramic vs. Ceramic-on-PE** | | | | |
| 5 yrs | 1/166 vs. 1/146^38^ | p=0.797 (NS); RR= 0.87, 95% CI: 0.05, 13.93^£^ | 1 [low ROB] | Inconclusive |
| **Femoral head-on-cup liner bearing surfaces-II**  **Ceramic-on-Ceramic vs. Metal-on-PE** | | | | |
| 10 yrs | 3/222 vs. 15/106^28^ | p<0.001 (SS); RR=0.10, 95% CI: 0.02, 0.32^£^ | 1 [low ROB] | In favour of Ceramic-on- Ceramic bearing surface |
| **Femoral stem composition**  **CoCr vs. Titanium** | | | | |
| 5 yrs | 0/199 vs. 0/191^40^ | p=NR; RR and 95% CI not estimated | 1 [unclear ROB] | Inconclusive |
| **Femoral stem fixation**  **Cemented vs. Cementless** | | | | |
| 20 yrs | Acetabular:  35/109 vs. 40/110^42^  Femoral:  31/109 vs. 35/110^42^ | p=0.168 (NS); RR=0.88, 95% CI: 0.61, 1.27^£^  p=0.159 (NS); RR=0.89, 95% CI: 0.59, 1.33^£^ | 1 [low ROB] | Inconclusive |
| RR=risk ratio (relative risk); SROB=summary risk of bias; SD=standard deviation; 95% CI=95 percent confidence interval; NR=not reported; SS=statistically significant; NS=statistically not significant; mo=month(s); yr(s)=year(s); PE=polyethylene; HA=hydroxyapatite; CoCr= cobalt chrome; HXLPE=highly cross-linked polyethylene; XLPE= cross-linked polyethylene | | | | |

Only those comparisons listed for which any evidence for the given outcome was reported

* Favours THR-1 (or THR-2), no difference, or inconclusive

** Decision was consensus-based

^£^ Calculated

**Appendix 17:**

**Table S15.**

**Risk of aseptic loosening (n/N)**

| **Follow-up** | **Arm-specific estimates**  **n/N or mean (SD or 95% CI)** | **Difference**  **(p value or 95% CI)** | **# of RCTs**  **[SROB across studies]**** | **Treatment effect**  **Conclusion*** |
| --- | --- | --- | --- | --- |
| **Cup fixation**  **Cemented vs. Cementless** | | | | |
| 10 yrs | 11/183 vs. 2/104^24^ | p=NR; RR= 3.12, 95% CI: 0.70, 13.83^£^ | 1 [low ROB] | Inconclusive |
| **Cup liner bearing surface**  **XLPE vs. Non XLPE** | | | | |
| 10 yrs | 0/111 vs. 0/109^26^ | NA; RR and 95% CI not estimated | 1 [unclear ROB ] | Inconclusive |
| **Cup and femoral stem fixation**  **Cemented cup/femoral stem vs. Cementless cup/femoral stem** | | | | |
| 20 yrs | 9/124 vs. 4/126^32^ | p=NR; RR= 2.28, 95% CI: 0.72, 7.23^£^ | 1 [low ROB] | Inconclusive |
| **Femoral head bearing surface**  **Oxinium femoral heads vs. CoCr femoral heads** | | | | |
| 2 yrs | 0/50 vs. 1/50^37^ | p=NR; RR and 95% CI not estimated | 1 [low ROB] | Inconclusive |
| **Femoral stem composition**  **CoCr vs. Titanium** | | | | |
| 5 yrs | 1/199 vs. 0/191^40^ | p=0.324 (NS); RR and 95% CI not estimated | 1 [unclear ROB] | Inconclusive |
| RR=risk ratio (relative risk); SROB=summary risk of bias; SD=standard deviation; 95% CI=95 percent confidence interval; NR=not reported; SS=statistically significant; NS=statistically not significant; mo=month(s); yr(s)=year(s); PE=polyethylene; HA=hydroxyapatite; CoCr= cobalt chrome; HXLPE=highly cross-linked polyethylene; XLPE= cross-linked polyethylene | | | | |

Only those comparisons listed for which any evidence for the given outcome was reported

* Favours THR-1 (or THR-2), no difference, or inconclusive

** Decision was consensus-based

^£^ Calculated

**Appendix 18:**

**Table S16.**

**Risk of femoral fracture (n/N)**

| **Follow-up** | **Arm-specific estimates**  **n/N or mean (SD or 95% CI)** | **Difference**  **(p value or 95% CI)** | **# of RCTs**  **[SROB across studies]**** | **Treatment effect**  **Conclusion*** |
| --- | --- | --- | --- | --- |
| **Cup liner bearing service**  **XLPE vs. Non XLPE** | | | | |
| 10 yrs | 2/111 vs. 0/109^26^ | p=NR; RR and 95% CI not estimated | 1 [unclear ROB] | Inconclusive |
| **Cup shell design**  **Porous-coated shell vs. Arc-deposited HA-coated shell** | | | | |
| 10 yrs | 0/113 vs. 0/109^28^ | NA; RR and 95% CI not estimated | 1 [low ROB] | Inconclusive |
| **Femoral stem composition**  **CoCr vs. Titanium** | | | | |
| 5 yrs | 0/199 vs. 1/191^40^ | p=0.309 (NS); RR and 95% CI not estimated | 1 [unclear ROB] | Inconclusive |
| RR=risk ratio (relative risk); SROB=summary risk of bias; SD=standard deviation; 95% CI=95 percent confidence interval; NR=not reported; SS=statistically significant; NS=statistically not significant; mo=month(s); yr(s)=year(s); PE=polyethylene; HA=hydroxyapatite; CoCr= cobalt chrome; HXLPE=highly cross-linked polyethylene; XLPE= cross-linked polyethylene | | | | |

Only those comparisons listed for which any evidence for the given outcome was reported

* Favours THR-1 (or THR-2), no difference, or inconclusive

** Decision was consensus-based

**Appendix 19:**

**Table S17.**

**Risk of infection (n/N)**

| **Follow-up** | **Arm-specific estimates**  **n/N or mean (SD or 95% CI)** | **Difference**  **(p value or 95% CI)** | **# of RCTs**  **[SROB across studies]**** | **Treatment effect**  **Conclusion*** |
| --- | --- | --- | --- | --- |
| **Cup fixation**  **Cemented vs. Cementless** | | | | |
| 10 yrs | 0/183 vs. 2/104^24^ | p=NR; RR and 95% CI not estimated | 1 [low ROB] | Inconclusive |
| **Femoral head bearing surface**  **Oxinium femoral heads vs. CoCr femoral heads** | | | | |
| 2 yrs | 1/50 vs. 1/50^37^ | p=NR; RR= 1.00, 95% CI: 0.06, 15.55^£^ | 1 [low ROB] | Inconclusive |
| **Femoral head-on-cup liner bearing surfaces-I**  **Ceramic-on-Ceramic vs. Ceramic-on-PE** | | | | |
| 5 yrs | Superficial: 6/166 vs. 3/146^38^  Deep: 1/166 vs. 2/146^38^ | p=0.357 (NS); RR= 1.75, 95% CI: 0.44, 6.90^£^  p=0.909 (NS); RR= 0.43, 95% CI: 0.04, 4.79^£^ | 1 [low ROB] | Inconclusive |
| **Femoral head-on-cup liner bearing surfaces–II**  **Ceramic-on-Ceramic vs. CoCr-on-XLPE** | | | | |
| 4 yrs | Deep: 0/78 vs. 0/72^43^ | p=1.0 (NS); RR and 95% CI not estimated | 1 [low ROB] | Inconclusive |
| **Femoral stem composition**  **CoCr vs. Titanium** | | | | |
| 5 yrs | 1/199 vs. 0/191^40^ | p=0.324 (NS); RR and 95% CI not estimated | 1 [unclear ROB] | Inconclusive |
| RR=risk ratio (relative risk); SROB=summary risk of bias; SD=standard deviation; 95% CI=95 percent confidence interval; NR=not reported; SS=statistically significant; NS=statistically not significant; mo=month(s); yr(s)=year(s); PE=polyethylene; CoCr= cobalt chrome | | | | |

Only those comparisons listed for which any evidence for the given outcome was reported

* Favours THR-1 (or THR-2), no difference, or inconclusive

** Decision was consensus-based

^£^ Calculated

**Appendix 20:**

**Table S18.**

**Risk of deep vein thrombosis (n/N)**

| **Follow-up** | **Arm-specific estimates**  **n/N or mean (SD or 95% CI)** | **Difference**  **(p value or 95% CI)** | **# of RCTs**  **[SROB across studies]**** | **Treatment effect**  **Conclusion*** |
| --- | --- | --- | --- | --- |
| **Femoral head-on-cup liner bearing surfaces-I**  **Ceramic-on-Ceramic vs. Ceramic-on-PE** | | | | |
| 5 yrs | 3/166 vs. 2/146^38^ | p=0.909 (NS); RR= 1.31, 95% CI: 0.22, 7.78^£^ | 1 [low ROB] | Inconclusive |
| **Femoral head-on-cup liner bearing surfaces–II**  **Ceramic-on-Ceramic vs. CoCr-on-XLPE** | | | | |
| 4 yrs | 0/78 vs. 1/72^43^ | p=0.29 (NS); RR and 95% CI not estimated | 1 [low ROB] | Inconclusive |
| RR=risk ratio (relative risk); SROB=summary risk of bias; SD=standard deviation; 95% CI=95 percent confidence interval; NR=not reported; SS=statistically significant; NS=statistically not significant; mo=month(s); yr(s)=year(s); PE=polyethylene | | | | |

Only those comparisons listed for which any evidence for the given outcome was reported

* Favours THR-1 (or THR-2), no difference, or inconclusive

** Decision was consensus-based

^£^ Calculated

**Appendix 21:**

**Table S19.**

**GRADE evidence profile for gradable outcomes reported in randomized controlled trials (adapted from Guyatt et al., 2011)^19^**

| **Outcome**  **[follow-up timing]** | **N of studies reporting outcome (participants)** | **Pooled effect estimate**  **[95% CI] and conclusion** | **SROB across studies** | **Consistency** | **Directness** | **Precision** | **Outcome reporting bias** | **Quality of the evidence (GRADE)*** |
| --- | --- | --- | --- | --- | --- | --- | --- | --- |
| **Cup fixation (cemented vs. cementless) – 2 RCTs^22;24^** | | | | | | | | |
| **Harris Hip score**  **[6 mo-10 yrs]** | 2 (502) | None  **No difference** | Unclear | Consistent | Direct | Precise | Unlikely | Moderate |
| **WOMAC score [NA]** | 0 | NA | NA | NA | NA | NA | NA | NA (no evidence) |
| **Revision**  **[10 yrs]** | 1 (287) | None  **Inconclusive** | Low | NA | Direct | Imprecise | Likely | Very low |
| **Mortality**  **[10 yrs]** | 1 (215) | None  **Inconclusive** | Low | NA | Direct | Imprecise | Likely | Very low |
| **Femoral head penetration [NA]** | 0 | NA | NA | NA | NA | NA | NA | NA (no evidence) |
| **Implant dislocation**  **[10 yrs]** | 2 (502) | OR=0.34  95% CI: 0.13, 0.89  **In** **favour** of **cemented** **cup** | Low | Consistent | Direct | Precise | Unlikely | High |
| **Cup liner bearing surface (XLPE vs. Non XLPE) – 2 RCTs^25;26^** | | | | | | | | |
| **Harris Hip score**  **[1-10 yrs]** | 2 (320) | MD=2.29  95% CI:-0.88, 5.45  **No difference** | Unclear | Consistent | Direct | Precise | Unlikely | Moderate |
| **WOMAC score [1-5 yrs]** | 1 (100) | None  **No difference** | Unclear | NA | Direct | Precise | Likely | Very low |
| **Revision**  **[10 yrs]** | 1 (220) | None  **In favour of XLPE cup liner** | Unclear | NA | Direct | Precise | Likely | Very low |
| **Mortality**  **[5-10 yrs]** | 2 (320) | RR=1.39  95% CI: 0.78, 2.49  **Inconclusive** | Unclear | Consistent | Direct | Imprecise | Unlikely | Low |
| **Femoral head penetration [5-10 yrs]** | 2 (320) | None  **In favour of XLPE cup liner** | Unclear | Consistent | Direct | Precise | Unlikely | Moderate |
| **Implant dislocation [NA]** | 0 | NA | NA | NA | NA | NA | NA | NA (no evidence) |
| GRADE= Grading of Recommendations, Assessment, Development, and Evaluation; RCT=randomised controlled trial; CI=confidence interval; SROB=summary risk of bias; RCT=randomised controlled trial; NA=not applicable; yr(s)=year(s); mo(s)=month(s); THR=total hip replacement | | | | | | | | |

*GRADE categories: High, moderate, low, very low

**Appendix 22:**

**Table S20.**

**Characteristics of included systematic reviews**

| **Author (year)**  **Ref ID**  **Country** | **Study details** | **Search strategy** | **Inclusion criteria** | **Quality assessment** | **Methods of synthesis** |
| --- | --- | --- | --- | --- | --- |
| Voigt 2012^80^  US | ***Comparison category:*** Cup fixation  ***Aim:*** To compare uncemented metal backed acetabular components with polyethylene inserts to cemented all-polyethylene acetabular components (using the same type of femoral component and method of femoral fixation in both arms of trial) in terms of revision rates, function, complications, and costs in patients with osteoarthritis | ***Databases searched:*** PubMed-Medline, Cochrane Library  ***Last date of search:*** 13 June 2011 | ***Participants:*** Patients with osteoarthritis or rheumatoid arthritis  ***Interventions:*** Primary total hip implant with uncemented metal backed acetabular components with polyethylene inserts  ***Comparators:*** Primary total hip implant with cemented all-polyethylene acetabular components  ***Outcome measures:*** Revision rate, function (Harris Hip Score, Oxford Hip score), complications (infection or wound, deep vein thrombosis, pulmonary embolism, dislocations, overreaming, fractures, and costs of treatment)  ***Types of studies:*** RCTs | ***Quality assessment tool used:*** Cochrane risk of bias tool  ***Risk of bias assessment criteria:*** Sequence generation, allocation  concealment, blinding, incomplete outcome data, selective outcome reporting, and other issues  ***Summary of risk of bias of included studies:*** Random sequence generation (low=4/6 trials), allocation concealment (high=3/6 trials; low=2/6 trials), caregiver blinding (high=6/6 trials), patient blinding (unknown=4/6 trials; high=1/6 trials), assessor blinding (high=5/6 trials), incomplete outcome data (low=5/6 trials), selective reporting (low=6/6 trials), other bias (low=3/6 trials; high=2/6 trials) | **Direct comparison**  *a) Non-quantitative:* No  *b) Quantitative :* Yes  **Indirect comparison**  *a) Unadjusted:* No  *b) Adjusted:* No  *c) Mixed treatment comparison :* No  **Specific methods of assessment**  *a) Heterogeneity:* Yes  *b) Publication bias:* Yes  *c) Overall quality/strength of evidence (GRADE):* No |
| Pakvis 2011^45^  The Netherlands | ***Comparison category:*** Cup fixation  ***Aim:*** To identify all relevant RCTs and comparative cohort studies in which cemented and cementless sockets were compared | ***Databases searched:*** Medline and Embase (1980 - December 2009)  ***Last date of search:*** December 2009 | ***Participants:*** Indication for performing THA had to be primary or secondary osteoarthritis  ***Interventions:*** Cemented acetabular components  ***Comparators:*** Cementless acetabular components  ***Outcome measures:*** Minimal follow-up had to be 12 months; data presented had to be clinical (complications, Harris hip score and survival) and radiological outcome measurements (wear, migration and osteolysis)  ***Types of studies:*** Non-randomised studies; RCTs | ***Quality assessment tool used:*** van Tulder checklist (for RCTs); Newcastle-Ottawa quality assessment scale (for non-RCTs)  ***Risk of bias assessment criteria:*** van Tulder checklist (randomisation, allocation concealment, prognostic factors, patient blinding, surgeon blinding, outcome assessor blinding, co-interventions, compliance, drop-out, timing of the outcome assessments, intention to treat, and homogeneity); Newcastle-Ottawa quality assessment scale (representativeness of the exposed cohort, selection of the non-exposed cohort, ascertainment of exposure, demonstration that outcome of interest was not present at start of study, comparability of cohorts on the basis of the design or analysis, assessment of outcome, and adequacy of follow-up of cohorts)  ***Summary of risk of bias of included studies:*** There were three RCT studies which scored ‘yes’ on more than 50% of the van Tulder criteria. In orthopaedic surgery, surgeon blinding is not feasible. Therefore when re-evaluating the results of the van Tulder questionnaire we could select seven articles that scored yes on more than 50% of the van Tulder items | **Direct comparison**  *a) Non-quantitative:* Yes  *b) Quantitative :* No  **Indirect comparison**  *a) Unadjusted:* No  *b) Adjusted:* No  *c) Mixed treatment comparison :* No  **Specific methods of assessment**  *a) Heterogeneity:* No  *b) Publication bias:* No  *c) Overall quality/strength of evidence (GRADE):* No |
| Clement 2012^46^  France | ***Comparison category:*** Cup fixation  ***Aim:*** To perform a critical analysis of the current evidence from a systemic literature review of comparative studies, long-term case series, prior literature reviews, meta-analysis, and national arthroplasty registry data for cemented and uncemented acetabular components | ***Databases searched:*** Medline  ***Last date of search:*** 2011 | ***Participants:*** Young patients and for patients with dysplastic hip disease  ***Interventions:*** Cemented THR  ***Comparators:*** Uncemented THR  ***Outcome measures:*** Aseptic loosening, radiographic loosening, overall survival, wear rates per year, dislocation, osteolysis, acetabular revision, liner exchange, quality of life  ***Types of studies:*** 1) All published review articles and meta-analysis; 2) All studies comparing cemented with uncemented acetabular components with a minimal follow-up of 5 years, quoting survival and wear or complications; 3) All arthroplasty registers reporting a comparison between cemented with uncemented acetabular components; 4) Single centre outcome studies with more than 13 years; 5) Single centre series reporting survivorship of cemented or uncemented cups in young patients and for patients with dysplastic hip disease | ***Quality assessment tool used:*** None  ***Risk of bias assessment criteria:*** None  ***Summary of risk of bias of included studies:*** NA | **Direct comparison**  *a) Non-quantitative:* Yes  *b) Quantitative :* No  **Indirect comparison**  *a) Unadjusted:* No  *b) Adjusted:* No  *c) Mixed treatment comparison :* No  **Specific methods of assessment**  *a) Heterogeneity:* No  *b) Publication bias:* No  *c) Overall quality/strength of evidence (GRADE):* No |
| Sedrakyan 2011^81^  US | ***Comparison category:*** Femoral head bearing-on-cup liner bearing  ***Aim:*** To determine comparative safety and effectiveness of combinations of bearing surfaces of hip implants | ***Databases searched:*** Medline, Embase, and the Cochrane Controlled Trials Register from  January 1995  ***Last date of search:*** June 2011 | ***Participants:*** Adults enrolled, reporting any one of the clinical outcomes of interest (any functional outcomes or revisions, or both)  ***Interventions:*** Conventional hip replacement  ***Comparators:*** Conventional hip replacement  ***Outcome measures:*** Any functional outcome (Harris hip score and general quality of life measures such as SF-12) and occurrence of revision ***Types of studies:*** RCTs, controlled clinical trials, observational comparative controlled studies | ***Quality assessment tool used:*** Selected validity items (RCTs) and STROBE (observational studies)  ***Risk of bias assessment criteria:*** RCTs (methods of random allocation generation, allocation concealment, masking of patients and outcome assessors, and intention to treat analysis)  ***Summary of risk of bias of included studies:*** Four studies were classified as moderate to high quality, five studies as moderate quality, and six studies as low quality | **Direct comparison**  *a) Non-quantitative:* No  *b) Quantitative :* Yes  **Indirect comparison**  *a) Unadjusted:* No  *b) Adjusted:* No  *c) Mixed treatment comparison :* No  **Specific methods of assessment**  *a) Heterogeneity:* Yes  *b) Publication bias:* Yes  *c) Overall quality/strength of evidence (GRADE):* No |
| Yoshitomi 2009^48^  Japan | ***Comparison category:*** Femoral head bearing-on-cup liner bearing  ***Aim:*** To compare the survivorship/revision and annual PE wear rates between THRs with zirconia-on-polyethylene (PE) and nonzirconia-on-PE; to explore if manufacturers or fixation method influenced survivorship | ***Databases searched:*** PubMed (1966 to July 2007), EMBASE (1974 to July 2007), and the Cochrane Central Register of Controlled Trials (Issue 4, July 2007)  ***Last date of search:*** July 2007 | ***Participants:*** NR  ***Interventions:*** THA using zirconia heads with PE cup liner (regardless of femoral head size, method of fixation)  ***Comparators:*** THA using nonzirconia heads with PE cup liner (regardless of femoral head size, method of fixation)  ***Outcome measures:*** Survivorship/revision, PE wear rates  ***Types of studies:*** RCTs, nonrandomized controlled trials, and cohort studies with follow-up of > 5 years | ***Quality assessment tool used:*** The Cochrane Back Review Group 11-item criteria  ***Risk of bias assessment criteria:*** Generation of random allocation, allocation concealment, blinding, co-interventions, compliance, sample attrition, outcome assessment timing, and type of analysis  ***Summary of risk of bias of included studies:*** The mean (range) score for the cohort studies= 4.5 (4–5) for the RCTs= 6.3 (6–7) | **Direct comparison**  *a) Non-quantitative:* No  *b) Quantitative :* Yes  **Indirect comparison**  *a) Unadjusted:* No  *b) Adjusted:* No  *c) Mixed treatment comparison :* No  **Specific methods of assessment**  *a) Heterogeneity:* Yes  *b) Publication bias:* Yes  *c) Overall quality/strength of evidence (GRADE):* No |

**Appendix 23:**

**Table S21.**

**Methodological quality of systematic reviews (AMSTAR items)**

| **First author, year, study ID** | Was an ‘a priori’ design provided? | Was there duplicate study selection and data extraction? | Was a comprehensive literature search performed? | Was the status of publication (i.e. grey literature) used as an inclusion criterion? | Was a list of studies (included and excluded) provided? | Were the characteristics of the included studies provided? | Was the scientific quality of the included studies assessed and documented? | Was the scientific quality of the included studies used appropriately in formulating conclusions? | Were the methods used to combine the findings of studies appropriate? | Was the likelihood of publication bias assessed? | Was the conflict of interest stated? | **Overall** |
| --- | --- | --- | --- | --- | --- | --- | --- | --- | --- | --- | --- | --- |
| Voigt 2012^44^ | Yes | Yes | Yes | CA | Yes | Yes | Yes | Yes | Yes | Yes | Yes | High  quality |
| Pakvis 2011^45^ | Yes | No | Yes | Yes | No | Yes | Yes | No | No | No | No | Medium quality |
| Clement 2012^46^ | Yes | No | No | Yes | Yes | Yes | No | No | No | No | No | Low  quality |
| Sedrakyan 2011^47^ | Yes | Yes | Yes | No | Yes | Yes | Yes | Yes | Yes | Yes | No | High  quality |
| Yoshitomi 2009^48^ | Yes | Yes | Yes | CA | Yes | Yes | Yes | NA | No | Yes | No | Medium quality |
| ID=identification; NA= Not applicable; CA= Can’t answer | | | | | | | | | | | | |

The methodological quality of each systematic review was rated according to the number of items satisfied: high (range: 9-11), medium (range: 5-8), and low (range: 0-4)

**Appendix 23:**

**Table S22.**

*NB - Tables 22-29 (results for each outcome reported in systematic reviews)*

**Harris Hip score (range: 0-100)**

| **Follow-up** | **Pooled effect estimate (95% CI)** | **# of RCTs in MA or narrative synthesis** | **AMSTAR rating** | **Treatment effect**  **Conclusion*** |
| --- | --- | --- | --- | --- |
| **Cup fixation**  **Cemented vs. Cementless** | | | | |
| 3 yrs  2-5 yrs | NR ^44^  NR^45^ | 2^44^  3^45^ | High quality ^44^  Low quality ^45^ | Inconclusive |
| **Femoral head-on-cup liner surfaces-I**  **Metal-on-Metal vs. Metal-on-PE** | | | | |
| 2 yrs  >2yrs | MD=-2.40, 95% CI: -4.47, -0.33 (SS)^47^  MD=1.21, 95% CI: -2.41, 4.83 (NS)^47^ | 4^47^  2^47^ | High quality ^47^ | Inconclusive |
| **Femoral head-on-cup liner surfaces-II**  **Ceramic-on-Ceramic vs. Ceramic-on-PE** | | | | |
| NR | NR^47^ | 5^47^ | High quality ^47^ | Inconclusive |
| **Femoral head-on-cup liner surfaces-III**  **Ceramic-on-PE vs. Metal-on-PE** | | | | |
| NR | NR^47^ | 2^47^ | High quality ^47^ | Inconclusive |
| **Femoral head-on-cup liner surfaces-IV**  **Metal-on-Metal vs. Ceramic-on-Ceramic** | | | | |
| NR | NR^47^ | 1^47^ | High quality ^47^ | Inconclusive |
| MD=mean difference; 95% CI=95 percent confidence interval; NR=not reported; yr(s)=year(s); PE=polyethylene; SS=statistically significant; NS=statistically not significant; MA=meta-analysis | | | | |

Only those comparisons listed for which any evidence for the given outcome was reported

* Favours THR-1 (or THR-2), no difference, or inconclusive

**Appendix 24:**

**Table S23.**

**Oxford Hip score (range: 0-48)**

| **Follow-up** | **Pooled effect estimate (95% CI)** | **# of RCTs in MA or narrative synthesis** | **AMSTAR rating** | **Treatment effect**  **Conclusion*** |
| --- | --- | --- | --- | --- |
| **Cup fixation**  **Cemented vs. Cementless** | | | | |
| 3 yrs | NR ^44^ | 1^44^ | High quality ^44^ | Inconclusive |
| MD=mean difference; 95% CI=95 percent confidence interval; NR=not reported; yr(s)=year(s); PE=polyethylene; SS=statistically significant; NS=statistically not significant; MA=meta-analysis | | | | |

Only those comparisons listed for which any evidence for the given outcome was reported

* Favours THR-1 (or THR-2), no difference, or inconclusive

**Appendix 25:**

**Table S24.**

**Short Form Health Survey (SF-12; range: 0-100)**

| **Follow-up** | **Pooled effect estimate (95% CI)** | **# of RCTs in MA or narrative synthesis** | **AMSTAR rating** | **Treatment effect**  **Conclusion*** |
| --- | --- | --- | --- | --- |
| **Femoral head-on-cup liner surfaces-I**  **Metal-on-Metal vs. Metal-on-PE** | | | | |
| 2-3 yrs | NR^47^ | 2^47^ | High quality ^47^ | Inconclusive |
| MD=mean difference; 95% CI=95 percent confidence interval; NR=not reported; yr(s)=year(s); PE=polyethylene; SS=statistically significant; NS=statistically not significant; MA=meta-analysis | | | | |

Only those comparisons listed for which any evidence for the given outcome was reported

* Favours THR-1 (or THR-2), no difference, or inconclusive

**Appendix 26:**

**Table S25.**

**Risk of revision (n/N)**

| **Follow-up** | **Pooled effect estimate (95% CI)** | **# of RCTs in MA or narrative synthesis** | **AMSTAR rating** | **Treatment effect**  **Conclusion*** |
| --- | --- | --- | --- | --- |
| **Cup fixation**  **Cemented vs. Cementless** | | | | |
| 4-8 yrs  10 yrs  <10 yrs  5-15 yrs | RR=0.15, 95% CI: 0.02, 1.18 (NS)^44^  RR=1.36, 95% CI: 0.81, 1.29 (NS)^44^  NR^45^  NR^46^ | 2^44^  2^44^  6^45^  NR^46^ | High quality ^44^  Low quality ^45^  Low quality ^46^ | Inconclusive |
| **Femoral head-on-cup liner surfaces-I**  **Metal-on-Metal vs. Metal-on-PE** | | | | |
| 2-5 yrs | NR^47^ | 2^47^ | High quality ^47^ | Inconclusive |
| **Femoral head-on-cup liner surfaces-II**  **Ceramic-on-Ceramic vs. Metal-on-PE** | | | | |
| 6-8 yrs | NR^47^ | 1^47^ | High quality ^47^ | Inconclusive |
| **Femoral head-on-cup liner surfaces-III**  **Ceramic-on-Ceramic vs. Ceramic-on-PE** | | | | |
| 2-8 yrs | NR^47^ | 5^47^ | High quality ^47^ | Inconclusive |
| **Femoral head-on-cup liner surfaces-IV**  **Ceramic-on-PE vs. Metal-on-PE** | | | | |
| 8 yrs | NR^47^ | 1^47^ | High quality ^47^ | Inconclusive |
| **Femoral head-on-cup liner surfaces-V**  **Zirconia-on-PE vs. Non Zirconia-on-PE** | | | | |
| 9 yrs | RD=0.02, 95% CI: -0.01, 0.06 (NS)^48^ | 3^48^ | Medium quality ^48^ | No difference |
| 95% CI=95 percent confidence interval; NR=not reported; yr(s)=year(s); PE=polyethylene; RD=risk difference; SS=statistically significant; NS=statistically not significant; MA=meta-analysis | | | | |

Only those comparisons listed for which any evidence for the given outcome was reported

* Favours THR-1 (or THR-2), no difference, or inconclusive

**Appendix 27:**

**Table S26.**

**Implant survival rate (%)**

| **Follow-up** | **Pooled effect estimate (95% CI)** | **# of RCTs in MA or narrative synthesis** | **AMSTAR rating** | **Treatment effect**  **Conclusion*** |
| --- | --- | --- | --- | --- |
| **Cup fixation**  **Cemented vs. Cementless** | | | | |
| 2-6 yrs  3-15 yrs | NR ^45^ narrative synthesis stated that there was no significant difference in survival rates  NR^46^ pooled data from 11 comparative studies (most non-RCTs) presented only graphically concluded that cemented cup had better survival rate compared to cementless cup | 3^45^  NR^46^ | Low quality ^45^  Low quality^46^ | Inconclusive |
| MD=mean difference; 95% CI=95 percent confidence interval; NR=not reported; yr(s)=year(s); PE=polyethylene; SS=statistically significant; NS=statistically not significant; MA=meta-analysis | | | | |

Only those comparisons listed for which any evidence for the given outcome was reported

* Favours THR-1 (or THR-2), no difference, or inconclusive

**Appendix 28:**

**Table S27.**

**Risk of implant dislocation (n/N)**

| **Follow-up** | **Pooled effect estimate (95% CI)** | **# of RCTs in MA or narrative synthesis** | **AMSTAR rating** | **Treatment effect**  **Conclusion*** |
| --- | --- | --- | --- | --- |
| **Cup fixation**  **Cemented vs. Cementless** | | | | |
| 5-15 yrs | 12/914 (1.3%) vs. 28/696 (4.1%), p = 0.001^46^ Pooled data from nine comparative studies (most non-RCTs) suggested that cemented cups had lower dislocation rate compared to cementless cups | NR^46^ | Low quality ^46^ | Inconclusive |
| **Femoral head-on-cup liner surfaces**  **Metal-on-Metal vs. Metal-on-PE** | | | | |
| 2-5 yrs | NR^47^ No significant difference based on results from three RCTs | 3^47^ | High quality ^47^ | Inconclusive |
| 95% CI=95 percent confidence interval; NR=not reported; yr(s)=year(s); PE=polyethylene; MA=meta-analysis | | | | |

Only those comparisons listed for which any evidence for the given outcome was reported

* Favours THR-1 (or THR-2), no difference, or inconclusive

**Appendix 29:**

**Table S28.**

**Risk of osteolysis (n/N)**

| **Follow-up** | **Pooled effect estimate (95% CI)** | **# of RCTs in MA or narrative synthesis** | **AMSTAR rating** | **Treatment effect**  **Conclusion*** |
| --- | --- | --- | --- | --- |
| **Cup fixation**  **Cemented vs. Cementless** | | | | |
| 2-6 yrs  5-15 yrs | NR^45^  The analysis and narrative synthesis of RCT data showed no statistically significant difference in the occurrence of osteolysis between cemented and cementless cups.  NR^46^  Narrative synthesis of nine comparative studies (most non-RCTs) indicated lower rates of osteolysis in cemented cups. | 3^45^  NR^46^ | Low quality ^45^  Low quality ^46^ | Inconclusive  Inconclusive |
| 95% CI=95 percent confidence interval; NR=not reported; yr(s)=year(s); MA=meta-analysis | | | | |

Only those comparisons listed for which any evidence for the given outcome was reported

* Favours THR-1 (or THR-2), no difference, or inconclusive

**Appendix 30:**

**Table S29.**

**Risk of aseptic loosening (n/N)**

| **Follow-up** | **Pooled effect estimate (95% CI)** | **# of RCTs in MA or narrative synthesis** | **AMSTAR rating** | **Treatment effect**  **Conclusion*** |
| --- | --- | --- | --- | --- |
| **Cup fixation**  **Cemented vs. Cementless** | | | | |
| 5-15 yrs | NR^46^  Pooled data from 11 comparative studies (most non-RCTs) presented only graphically suggested higher rates of aseptic loosening with cemented vs. cementless cup. | NR^46^ | Low quality ^46^ | Inconclusive |
| 95% CI=95 percent confidence interval; NR=not reported; yr(s)=year(s); MA=meta-analysis | | | | |

Only those comparisons listed for which any evidence for the given outcome was reported

* Favours THR-1 (or THR-2), no difference, or inconclusive
